# Identification of the Biosynthetic Gene Cluster of New Piperazic Acid-Containing Lipopeptides with Cytotoxic Activity in the Genome of Marine Streptomyces PHM034

**DOI:** 10.3390/metabo13101091

**Published:** 2023-10-18

**Authors:** Ana Ceniceros, Librada Cañedo, Carmen Méndez, Carlos Olano, Carmen Schleissner, Carmen Cuevas, Fernando de la Calle, José A. Salas

**Affiliations:** 1 Departamento de Biología Funcional e Instituto Universitario de Oncología del Principado de Asturias (IUOPA), Universidad de Oviedo, 33006 Oviedo, Spain; acmedrano@alumni.unav.es (A.C.); cmendezf@uniovi.es (C.M.); olanocarlos@uniovi.es (C.O.); 2 Instituto de Investigación Sanitaria del Principado de Asturias (ISPA), 33006 Oviedo, Spain; 3 Drug Discovery Area, PharmaMar S.A. Avda. de los Reyes 1, Colmenar Viejo, 28770 Madrid, Spain; lcanedo@pharmamar.com (L.C.); ccuevas@pharmamar.com (C.C.); fdelacalle@pharmamar.com (F.d.l.C.); 4 Unolab Manufacturing, Avenida de las Flores 6, Humanes de Madrid, 28970 Madrid, Spain; cschleissner@unolab.es

**Keywords:** lipopeptides, cytotoxic activity, nonribosomal peptides

## Abstract

Three novel lipopeptides, PM130391 (1), PM130392 (2), and PM140293 (3) were obtained from cultures of *Streptomyces tuirus* PHM034 isolated from a marine sediment. Structural elucidation of the three compounds showed they belong to the nonribosomal peptides family, and they all contain an acylated alanine, three piperazic acids, a methylated glycine, and an N-hydroxylated alanine. The difference between the three compounds resides in the acyl chain bound to the alanine residue. All three compounds showed cytotoxic activity against human cancer cell lines. Genome sequence and bioinformatics analysis allowed the identification of the gene cluster responsible for the biosynthesis. Inactivation of a nonribosomal peptide synthase of this cluster abolished the biosynthesis of the three compounds, thus demonstrating the involvement of this cluster in the biosynthesis of these lipopeptides.

## 1. Introduction

According to the World Health Organization, cancer is currently the second leading cause of death in the world [1]. Early detection and appropriate treatments are essential for improving the prognosis of patients [2]. Treatments can vary from surgical removal, stem cell transplants, such as bone marrow transplants, and immunotherapy to improving the immune system’s response to cancerous cells or using chemical compounds (chemotherapy) or radiation (radiotherapy) to stop or slow down the growth of cancerous cells [3]. The first report on chemotherapeutic drugs was in 1946, using nitrogen mustard to treat tumors in blood-forming organs [4]. Another important problem that should be considered is that chemotherapy usually has severe side effects, like vomiting, anorexia, or anemia [5]. Therefore, it is of the utmost importance to find novel bioactive compounds that can overcome tumor resistance with less undesirable side effects.

Secondary metabolites (SMs), also known as specialized metabolites, are natural products that are not essential for the life of producing organisms but can give them an advantage during survival in unfavorable conditions. They are a wide variety of compounds with diverse molecular structures, which provide them with different activities and functions for producing organisms, such as protection from damage caused by radiation, the capture of scarce resources, or acting as toxins for other organisms [6,7]. However, in many cases, the natural function of SMs is unknown [8,9]. SMs have diverse uses for humans, from pigments to antibiotics or antitumor drugs [10,11,12,13,14]. Several compounds isolated from microorganisms are in clinical trials as cancer treatments, such as ulocladol, salinosporamide, or plinabulin [15,16]. Lipopeptides are SM that are composed of a hydrophilic moiety (peptide) and a hydrophobic moiety (an acyl chain). They have a wide variety of structures that depend on the amino acids that form the peptidic moiety and the acyl chain that can vary even in the same family of compounds [17,18]. This structural variation leads to a wide range of activities, from their pharmacological use as antibiotics such as daptomycin or chemotherapeutics in the food industry, cosmetics, and many other functions [18]. Actinomycetes have been one of the main sources of bioactive compounds used in medicine. With the fast development of sequencing and genomics, it has been observed that strains known for the production of one or a few molecules have the potential to produce many more unknown metabolites [10,11,19,20]. The *Streptomyces* species are a source of important antitumor compounds such as actinomycin, mitomycin, anthracycline, or bleomycin. Unfortunately, in recent decades, the rate of rediscovery of already-described molecules has frustrated many research efforts [21]. In order to find novel molecular structures, several different strategies are being followed, from manipulating the genome to forcing the expression of cryptic clusters (genome mining) [22] or looking for new strains in barely explored environments such as the Earth’s poles, insect teguments, high altitudes, caves or marine sources [20,23,24,25,26,27]. Several different cytotoxic compounds have been recently isolated from *Streptomyces* [28,29,30,31]

In this work, we isolated microorganisms from marine sediments in Indonesia. One of the strains isolated was a bacterium from the genus *Streptomyces*. *Streptomyces* is a Gram-positive filamentous genus that has a complex life cycle, producing vegetative mycelia and aerial mycelia, which form spores when the nutrients in the medium are limited. They have large genomes with a high GC content. They produce most of the natural bioactive compounds used today in medicine [10]. The isolated strain was sequenced and classified as *Streptomyces tuirus* PHM034. Here, we describe three novel lipopeptides 1, 2, and 3 produced by this strain that show cytotoxic activity, and we isolated and identified the gene cluster responsible for its biosynthesis. Evidence is also provided based on the involvement of the gene cluster in the biosynthesis of these lipopeptides. These compounds are lipopeptides that contain a piperazic acid motif in their cyclic hexaptide structure, a non-proteogenic amino acid synthesized from ornithine, and not structurally related to other lipopeptides reported from actinomycetes [32,33]. Natural products cyclopeptides and cyclodepsipeptides containing a piperazate residue are known to have diverse bioactivities, including cytotoxic effects, which makes them interesting subjects for further study [32,33].

## 2. Materials and Methods

### 2.1. Strains, Media, and Culture Conditions

All strains used in this work are listed in Table 1. *S. tuirus* PHM034 was isolated from marine sediment and was grown in Soy Flour Mannitol (SFM) for spore generation [34]. *Streptomyces* strains were kept in spore form in 50% glycerol stocks. For the production of 1, 2, and 3, *S. tuirus* was grown in a PhamaMar’s induction medium (see Section 2.4). For protoplast generation, *S. tuirus* PHM034 was grown in 50% TSB/YEME containing 20% glycine and 0.05% tween 20 to obtain a dispersed culture [34]. After protoplast transformation, R5 media was used for regeneration [34]. The commercial strain *Escherichia coli* DH5α was used as a cloning strain and grown in 2YT [34]. When required, apramycin was added to the media as a selection method at a final concentration of 50 µg/mL for both *Streptomyces* and *E. coli* strains.

### 2.2. Isolation of the Strain

*Streptomyces tuirus* PHM034 was isolated from marine sediment collected at a 45 m depth in the Indian Ocean (1°59.618′ S 99°34.786′ E) in October 2010. The sample was stored in frozen conditions with glycerol 30% for one week until arrival at the laboratory. Then, a suspension of the sediment was spread on nutrient agar plates of the following composition (g/L): L-asparagine, 2.5; glycerol, 20; NaCl, 5.34; KCl, 0.15; Mg_2_SO_4_ × 7H_2_O, 0.1; FeSO_4_ × 7H_2_O, 0.12; Na_2_SO_4_, 7.5; MgCl_2_ × 6H_2_O, 2.4; CaCO_3_, 0.1; agar, 20; supplemented with trace elements (µg/L): CoCl_2_ × 6H_2_O, 40; CuSO_4_ × 5H_2_O, 40; ZnCl_2_ × 7H_2_O, 200; AlCl_3_ × 6H_2_O, 12; Na_2_MoO_4_ × 2H_2_O, 50; H_3_BO_3_, 200; K_2_CrO_4_, 12; SeO_3_H_2_, 10; NaF, 25; KBr, 5; Ni_2_(SO_4_) × 6H_2_O, 10; V_2_O_4_, 5; amino acids solution (g/L): L-Phe, 0.2; L-Pro, 0.2; L-Tyr, 0.2; L-Trp, 0.2, vitamins solution (µg/L): thiamine (B1), 500; biotin, 50; cyanocobalamin (B12), 50; folic acid (B9), 2; riboflavin (B2), 200; nicotinic acid (B3), 400; pyridoxine (B6), 400; pantotenic acid (B5), myo-inositol; and supplemented with the antibiotics cycloheximide (0.71 mM) and nalidixic acid (0.79 mM). The plates were incubated at 28 °C for one month under atmospheric pressure. After this period of time, colonies were picked and isolated onto agar plates in an ATCC Medium 172 for taxonomy studies.

### 2.3. Taxonomy

A taxonomic evaluation of *Streptomyces tuirus* PHM034 was conducted by a partial sequence of 16S rRNA, following standard procedures [36]. The strain was grown in ATCC’s 172 solid medium supplemented with 1% artificial marine salt during days 4–5. Cells were recovered and lysed, boiling with NP40 4% for 10 min. Cell debris was discarded via centrifugation. The 16S rDNA gene was amplified by the polymerase chain reaction using the actinobacterial primers F1/R5 described by Cook A.E. and Myers P.R. [37] The partial sequence of 830 bp was confronted with the ARB_SILVADDBB (https://www.arb-silva.de/ (accessed on 23 May 2016) for type species. These results show that the strain belonged to the phylogenetic genus *Streptomyces tuirus* AB184690 99.52%.

### 2.4. Fermentation and Extraction

A seed culture was developed in two scale-up steps, firstly in 100 mL Erlenmeyer flasks containing 20 mL of the seed medium and then 250 mL Erlenmeyer flasks with 50 mL of the same medium. The seed culture was grown on a medium containing dextrose 0.1%, soluble starch 2.4%, soy peptone 0.3%, yeast extract 0.5%, Tryptone 0.5%, soya flour 0.5%, sodium chloride 0.54%, potassium chloride 0.02%, magnesium chloride 0.24%, sodium sulfate 0.75%, and calcium carbonate at 0.4% in tap water (Madrid) after the strain was cultivated for 3 days at 28 °C and 220 rpm. For production, 12.5 mL of the seed medium was transferred into 2 L Erlenmeyer flasks containing 250 mL of the fermentation medium (MP12) containing soy peptone 0.1%, soya flour 1.2%, dextrose 0.25%, malt extract 0.1%, dextrin 4%, artificial seawater 2% and calcium carbonate 0.8%; the culture was grown at 28 °C 220 rpm. In total, 120 flasks of 2 L containing 250 mL were performed to give a total of 30 L of culture. After 13 days of cultivation, the culture was centrifuged to separate the mycelial cake and other solids from the clarified broth. The supernatant (26 L) was extracted with 26 L of ethyl acetate. The organic phase was concentrated under a reduced pressure to give 5.31 g of the oily supernatant extract. The mycelial cake (980 g) was extracted with (1/2/3 *w*/*v*/*v*), isopropanol (2-PrOH), and ethyl acetate (EtOAc) to give a crude extract (15.6 g). Although the cell extract contained lipopeptides, the purified compounds were extracted from the supernatant extract.

### 2.5. Isolation and Structural Elucidation of 1, 2 and 3

The supernatant extract was applied to a reverse phase silica gel Polygoprep 100–50 C18 VFC (vacuum flash chromatography) system, using stepwise gradient elution from H_2_O to MeOH. The active fraction (2.72 g) was eluted with H_2_O-MeOH 1:9 and was subjected to preparative reversed-phase HPLC equipped with a Symmetry C18 column (19 × 150 mm, 7 μ) and using a linear gradient of H_2_O/CH_3_CN from 5% to 100% of CH_3_CN in 60 min at a flow rate of 15 mL/min. The active fractions were further purified by a semi-preparative HPLC on a Symmetry C18 column (7.8 × 150 mm, 7 μ) using isocratic elution with H_2_O/CH_3_CN 55:45 at a flow rate of 3 mL/min to yield 80 mg of the pure compound **1**, 94 mg of compound **2** and 44 mg of compound **3**. NMR spectra were recorded on a Varian “Unity 500” spectrometer at 500/125 MHz (^1^H/^13^C) and on a Varian Unity 400 spectrometer at 400/100 MHz (^1^H/^13^C). Chemical shifts were reported in ppm using residual CDCl_3_ (δ 7.26 ppm for ^1^H and 77.0 ppm for ^13^C) as an internal reference. Two-dimensional experiments COSY, TOCSY, HSQC, and HMBC were performed using standard pulse sequences. Data were processed using MestReNova 14.0.1 software. (+)-ESIMS spectra obtained on an Agilent 1100 Series LC/MSD spectrometer. High-Resolution Mass Spectroscopy (HRMS) was performed on an Agilent 6230 TOF LC/MS system using the ESIMS technique.

### 2.6. Sequencing of the Genome

*S. tuirus* PHM34 genomic DNA was extracted following the protocol described by Keiser et al., 2000 [34]. The genome was then sequenced at Macrogen INC. using the PacBio RSS sequencer. De novo assembly was performed using FALCON software, and errors were corrected using Quiver. This Whole Genome Shotgun project has been deposited at DDBJ/ENA/GenBank under the accession JAGTPG000000000. The version described in this paper is version JAGTPG010000000. Compounds **1**, **2**, and **3** of the biosynthesis gene cluster were loaded into the MIBiG database with accession number BGC0002121.

### 2.7. Bioinformatic Analysis

The sequence was analyzed to predict putative secondary metabolite clusters encoded in the genome of this strain. An analysis was made with two different programs, antiSMASH 6.0 [38] and PRISM 3 [39]. AntiSmash was run using relaxed detection strictness with all extra functions on.

### 2.8. Construction of Disruption Plasmid and Protoplast Generation and Transformation

All primers and their sequence are annotated in Table 2. Primers PM13 Dis FW and PM13 Dis Rv were designed to amplify a region of 2.5 kb situated inside the last module of the NRPS (Table 2). PCR was performed using a Phusion polymerase, following the instructions given by the manufacturer. This amplicon was then cloned in the suicide vector pOJ260 [35] using restriction enzymes XbaI and BamHI. Plasmid was transformed into protoplasts of *S. tuirus*, as described in the following section. Correct insertion was then checked via a PCR using the primer pairs PM13Dis1-M13 and PM13 Dis1 checK-pOJ260 We then checked annealing in the chromosome outside the homologous region and inside the plasmid at both sides of the insertion. A disruption construct was introduced in S. tuirus PHM34 through protoplast generation [34]. The strain was grown in TSB/YEME 50% with 20% glycine and tween 20 at 0.05% to avoid clump formation. The transformation of protoplasts was performed following the protocol in Keiser et al., 2000 [34]. Apramycin-resistant colonies were then re-plated in apramycin selective media to verify their resistance to this antibiotic. Resistant colonies were then grown for spore collection.

### 2.9. Bioactivity of Fermentation Broth

Samples from cultures: 2 mL from each culture were lyophilized and extracted with 3 mL of methanol/acetone/H_2_O (1:1:0.2). After extraction and centrifugation, 1 mL of the organic phase was dried and used to evaluate cytotoxic activity after ×4 replicated assays. The results are average, and, in all cases, the variability found was less than that allowed in the standard screening assay using positive (doxorubicin) and negative (DMSO 1%) controls after ×4 replicated assays. The results were average, and in all cases, the variability found was less than that allowed in the standard screening assay using positive (doxorubicin) and negative (DMSO 1%) controls, Table 3.

### 2.10. Citotoxic Activity Assays

For the antiproliferative experiments described for 1, four human tumor cell lines were used: A549 (ATCC CCL-185) (lung carcinoma, NSCLC); HT-29 (ATCC HTB-38) (colon adenocarcinoma); MDA-MB-231 (ATCC HTB-26) (breast adenocarcinoma) and PSN-1 (ATCC CRL-3211) (pancreas adenocarcinoma). All cell lines were obtained from the American Type Culture Collection (ATCC) and derived from different types of human cancer.

Cells were maintained in Dulbecco’s Modified Eagle Medium (DMEM) supplemented with 10% Fetal Bovine Serum (FBS), 2 mM of L-glutamine, 100 U/mL of penicillin, and 100 U/mL of streptomycin at 37 °C, 5% CO_2_ and 98% humidity. For the experiments, cells were harvested from subconfluent cultures using trypsinization and suspended in a fresh medium before counting and plating. Cells were seeded in 96 well microtiter plates, at 5000 cells per well in aliquots of 150 µL, and were allowed to attach to the plate surface for 18 h (overnight) in a drug-free medium. After that, one control (untreated) plate of each cell line was fixed and used for the time zero reference value. Culture plates were then treated with test compounds (50 µL aliquots of 4× stock solutions in complete culture medium plus 4% DMSO) using ten 2/5 serial dilutions (concentrations ranging from 10 to 0.003 µg/mL) and triplicate cultures (1% final concentration in DMSO). After 72 h of treatment, the effects on cell growth and survival were estimated by applying the NCI algorithm [41].

### 2.11. Antimicrobial Activity

Experiments to study the antimicrobial activity of 1, 2, and 3 against *Pseudomona fluorescens*, *Escherichia coli*, *Staphylococcus aureus*, and *Candida albicans* were carried out with concentrations of 1, 2, and 3 from 0 to 2 mg/mL. Cell growth was monitored by optical density at 630 nm after 72 h, 96 h, 144 h, 168 h, and 214 h of fermentation.

## 3. Results

### 3.1. Strain Identification

TYGS analysis of the full genome sequence of the strain provided a low % of dDDH (44.1 d_4_), probably due to sequencing errors [42]. Therefore, the strain was identified using a partial sequence of 16S RNA as *Streptomyces tuirus.*

### 3.2. Bacteria Cultivation and Identification of Lipopeptides 1, 2, and 3

**Figure 1:**
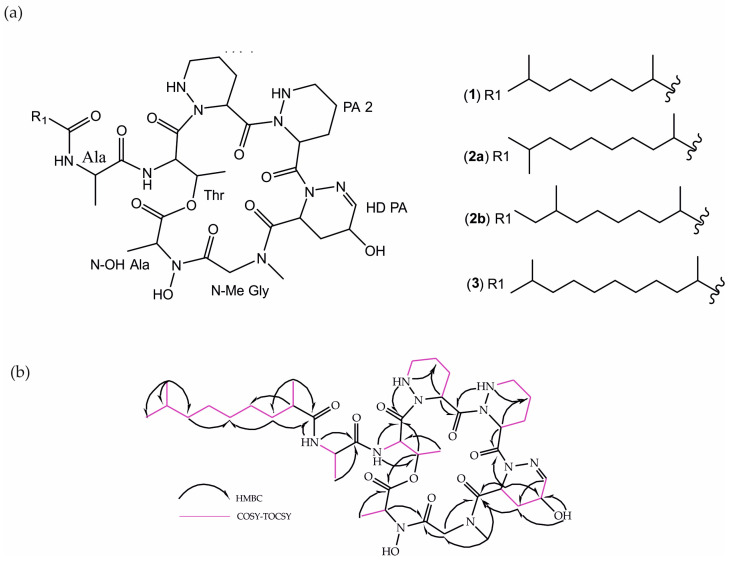
(**a**) Structure of PM130391 (**1**), PM130392 (**2a**, and **2b**) and PM140293 (**3**) (**b**) COSY, TOCSY, and HMBC correlations of compound **1**.

Cultures of the marine isolated *Streptomyces tuirus* PHM034 were scaled up and their crude extracts were fractionated via preparative HPLC to isolate three active compounds **1** to **3**, as shown in Figure 1a.

Compound **1**, with a yield of 80 mg/30 L (2.6 mg/L) was isolated as a colorless amorphous solid, and its molecular formula C_39_H_64_N_10_O_11_ was determined from its ESIMS *m*/*z* 849.3 [M + H]^+^ and 871.4 [M + Na]^+^; HRESIMS *m*/*z* 849.4853 [M + H]^+^ (calcd for C_39_H_65_N_10_O_11_, 849.4829) and 871.4676 [M + Na]^+^ (calcd for C_39_H_64_N_10_O_11_Na, 871.4648), in conjunction ^13^C NMR data, Figures S2 and S15. A preliminary examination of the 1D NMR spectra recorded for **1**, Table 4, revealed the presence of signals in the NH and α-amino acid proton regions in its ^1^H NMR and of carbonyl groups in the ^13^C NMR spectrum, which were indicative of a peptidic structure, Figures S1 and S2. Detailed analysis of the COSY, TOCSY, HSQC, and HMBC, and data, Figures S3–S6, identified the amino acid content of the peptide and the existence of one acyl chain corroborated by the aliphatic signals corresponding to CH_3_ and CH_2_ in the ^1^H and ^13^C NMR spectra, Table 4, Figures S1 and S2. The amino acid sequence and the position of the acyl chain connection were determined via HMBC correlations, as shown in Figure 1b. The saturated fatty acid residue was identified as 2,8-dimethyl nonanoic acid (DMNA) based on NMR data and the molecular formula established by HRMS.

The structural elucidation of **2** and **3** was mainly focused on **1**. The 1D and 2D NMR data of **2** and **3** were closely similar to those of **1** and revealed the same core structure of **1**, Figures S7–S14. However, MS data suggested that **2** and **3** differed in their fatty acid chains with **1**, Figures S16 and S17.

The molecular formula of compound **2**, with a yield of 94 mg/30 L (3.1 mg/L), was established as C_40_H_66_N_10_O_11_ via HRESIMS analysis at *m*/*z* 863.5055 [M + H]^+^ (calcd. for C_40_H_67_N_10_O_11_, 863.4985) and 885.4808 [M + Na]^+^ (calcd. for C_40_H_66_N_10_O_11_Na, 885.4805); compound **2** was 14 mass units higher than **1**, with one additional methylene, Figure S16. Detailed analysis of ^13^C NMR data indicated that compound **2** was a mixture of two peptides with the same molecular weight but different fatty acid chain, **2a** and **2b**, Figures S8 and S9; therefore, in the case of **2a**, the fatty acid chain was a 2,9-dimethyl decanoic acid (DMDA) and for **2b** a 2,8-dimethyl decanoic acid residue was identified.

Compound **3**, with a yield of 44 mg/30L (1.5 mg/L), had the molecular formula C_41_H_68_N_10_O_11_, as determined by HRESIMS analysis at *m*/*z* 877.5192 [M + H]^+^ (calcd. for C_41_H_69_N_10_O_11_, 877.5142) and 899.5034 [M + Na]+ (calcd. for C_41_H_68_N_10_O_11_Na, 899.4961) as well as ^13^C NMR data, Figures S14 and S17. Compound **3** was 28 mass units higher than **1**, with the existence of two additional methylene signals (δc 29.50 and 29.44), which were observed in its ^13^C NMR spectrum. Finally, the fatty acid residue was identified as 2,10-dimethyl undecanoic acid (DMUDA) based on NMR chemical shifts and the molecular formula established using HRMS.

### 3.3. In Vitro Bioactivity

The bioactivity of the three lipopeptides was performed in cytotoxic assays against cell lines of lung carcinoma A549 (ATCC CCL-185); colorectal carcinoma HT29 (ATCC HTB-38); breast adenocarcinoma MDA-MB-231 (ATCC HTB-26); and pancreas adenocarcinoma PSN1 (ATCC CRN-CRL-3211) cell lines. The cytotoxic activities in molar concentrations are summarized in Table 3. All three compounds showed strong cytotoxicity against all tested cell lines, the lowest being the activity of **1** against the cell line of lung carcinoma A549, which still showed GI_50_ of 100 nM. The highest cytotoxicity of all three compounds was observed against the cell line of pancreas adenocarcinoma PSN1, with **3** being the one with the highest activity and a GI_50_ of 3 nM, Table 3.

Compounds were also tested for antimicrobial activity, but no bioactivity was observed.

### 3.4. Genome Sequencing and Identification of Secondary Metabolism Gene Clusters

The *S. tuirus* PHM034 genome was sequenced into two contigs. This included Contig 1 (NCBI accession number JAGTPG010000002) of 6.57 Mb and Contig 2 (GenBank accession number JAGTPG010000001) of 2.44 Mb. It had a high guanine and cytosine content (71%), which is a common feature of actinomycetes. In order to isolate and identify the biosynthesis gene cluster (BGC) responsible for the biosynthesis of these compounds, antiSMASH 6.0 [38] and PRISM 3 [39] were used to analyze the sequence of *S. tuirus* PHM34 and to predict putative BGCs encoded in the genome. AntiSMASH predicted 27 different putative BGC (Table S1) and PRISM 21 clusters.

**Figure 2:**
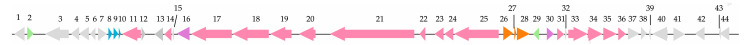
Putative BGCs responsible for the biosynthesis of **1**, **2**, and **3**. Genes in grey probably do not have a role in the biosynthesis of these lipopeptides. NRPS encoding genes are shown in red. PS genes are shown in orange. Regulators are shown in green. Transporters are shown in blue. The cluster encodes for KtzI and KtzT homologous enzymes that are essential to transform ornithine into piperazic acid (shown in purple color) [32]. Gene codification and predicted function f and the encoded enzymes are shown in Table S2.

Since the compounds were lipopeptides containing seven amino acids, we analyzed all NRPS containing seven modules and capable of adding an acyl chain to the NRP [43,44]. Therefore, we searched for clusters containing a condensation C-starter domain, a standalone fatty acid CoA ligase or synthetases, and/or hybrid NRPS/PKS clusters. The amino acids detected in the structure of these compounds were two alanine, three piperazic acids, one hydroxylate, a methylated glycine, and a threonine. Therefore, we looked for an NRPS cluster that was predicted to incorporate the observed residues. AntiSMASH cluster number 1 from contig 2 was identified as a possible candidate for the biosynthesis of the detected SM, Figure 2. PRISM predicted that this cluster incorporates three piperazic acid molecules. Apart from that, PRISM predicted the heme-dependent piperazate synthase homologs of KtzT, and antiSMASH predicted the L-ornithine N(5)-oxygenase (KtzI homolog) needed for the conversion of ornithine into piperazic acid [32]. This cluster also contained a seemingly stand-alone C-starter domain, which is known to introduce an acyl chain in the first amino acid. This could explain the acyl chain bound to an alanine found in **1**, **2**, and **3** that differentiates these three compounds. It also contains a cytochrome P450 that can be involved in the hydroxylation of piperazic acid and the last alanine of the compounds. The other two residues were predicted to be serine and valine, respectively. However, they were both found to be alanine in our structural elucidation PRISM also predicted a halogenase as part of the BGC, but we did not find any halogenation in the three lipopeptides. antiSMASH predicted an ACP-S-malonyltransferase, an ACP, and a beta-keto-[acyl-carrier-protein] synthase family protein. AntiSMASH prediction covered a bigger cluster with more biosynthetic genes than we expected. Based on the structure of these compounds, we believe they are not involved in the biosynthesis of compounds since the genes previously described already explain the full structure.

The first module of the NRPS was probably formed by the products of genes 31 to 36. This module contained a C-starter domain that could introduce the acyl chains present in the detected molecules. However, this module was predicted to introduce a valine while the amino acid incorporated in the structures elucidates was, in fact, an alanine.

In the gene cluster, there was also an MbtH-like protein encoded (gene 15), which are small protein known to be associated with adenylation domains and which is indispensable for the proper folding of the domain [45]. Some of these enzymes are known to be able to interact with more than one NRPS cluster, even enzymes from bacteria are known to stimulate NRP production in fungi [46].

### 3.5. Inactivation of the NRPS

To confirm that the putative-identified BCG was responsible for the biosynthesis of PM130391, 130392, and 140293, we performed a gene disruption experiment in the last module of the NRPS, preventing the release of the molecule from the enzyme through the thioesterase domain. Two clones were obtained (*S. tuirus* PM13 Dis7 and *S. tuirus* PM13 Dis8) and verified via PCR using primers inside the vector pOJ260 (used for disruption) and outside the homology zone to prove that the insertion took place in the correct site, Figure S18.

Extracts from both mutants, *S. tuirus* PM13 Dis7, *S. tuirus* PM13 Dis8, and from the wild-type strain were then tested for bioactivity and analyzed using LC/MS to check if the production of the compounds had been abrogated. In both mutants, the ability to produce the three lipopeptides was completely lost, as shown by LC/MS analysis, Figure S19.

## 4. Discussion

Microorganisms are the source of many chemically diverse natural products with different types of bioactivities [15]. Currently, several of them are in clinical trials as cancer treatments [15,16]. Actinomycetes have been an important supplier of bioactive compounds, and they are known to be able to produce many other metabolites that are currently cryptic or silent [19]. In this work, we found three novel lipopeptides with cytotoxic activity isolated from the marine strain *S. tuirus* PHM034, and we identified the gene cluster responsible for its biosynthesis. No antimicrobial activity was observed from these compounds. The lipopeptides **1**, **2**, and **3** were biosynthesized using a non-ribosomal peptide synthetase type gene cluster. Enzymes are responsible for introducing five of the amino acids detected during the structural elucidation of **1**, **2**, and **3** were predicted by PRISM during genome sequence analysis: three piperazic acids, a methylated glycine, and a threonine. The two alanine moieties found in the structures were predicted using PRISM as enzymes that added serine and valine. The prediction of residues introduced by NRPS adenylation domains is not always accurate.

Threonine seems to be the second amino acid residue added to the molecule since it was bound to the acylated alanine (presumably loaded by the enzymes codified by genes 31 to 36, Figure 2). However, it was introduced in the molecule through a separated enzyme from the main NRPS that contained only an adenylation domain and a thiolation domain (predicted by PRISM) (codified by 11, Figure 2). In an attempt to understand how this residue was added to the molecule, the NRPS gene cluster was carefully analyzed. Close to gene 11, there is a gene codifying a stand-alone thioesterase (gene 14 in Figure 2). A blast of this protein showed homology with thioesterases but also to an alpha/beta fold hydrolase. Shingh et al., 2007 described how SyrC alpha/beta hydrolase transports the amino acid threonine, which is loaded by a stand-alone enzyme containing an adenylation and thiolation domain and transports it for addition as the last amino acid in syringomycin [47]. Therefore, this could be the mechanism followed to introduce threonine in these compounds.

Another particularity of this biosynthetic gene cluster is that the C-starter domain predicted to introduce the acyl chain (gene 31, Figure 2) seems to be a standalone enzyme. We were unable to find previous reports of a standalone C-starter domain, which led us to consider that, most probably, genes 31 to 36 are a single NRPS gene, as indicated in Figure 2, and that sequencing errors are due to PacBio difficulties sequencing high GC content organisms [48]. We suggest that the acylated alanine is somehow bound to the threonine, and then both of the residues are bound to the last alanine added by the main NRPS. More experiments are needed to confirm this theory, such as knocking out the thioesterase/alpha/beta hydrolase. The predicted ACP-S-malonyltransferase, an ACP, and a beta-keto-[acyl-carrier-protein] synthase family protein (26, 27 and 28, Figure 2) may be responsible for the synthesis of acyl chains and loading into the C starter domain [49].

We also found in the gene cluster that a gene encoding a homolog of KtzI (gene 16, Figure 2), and a homolog of KtzT enzyme (gene 30, Figure 2), catalyze the transformation of ornithine into piperazic acid, which is the residue introduced by the NRPS [32]. There is also a cytochrome p450 (encoded by 8, 9, and 10, Figure 2) that might probably be involved in the hydroxylation of the alanine and piperazic acid residues found in this structure. An ABC transporter is also present in the gene cluster, most probably being the export system of the compounds, and it could also be the resistance system that *S. tuirus* PHM034 has for its own bioactive compounds [49]. With all these data, we could initially assume that this BGC is responsible for the biosynthesis of lipopeptides. An LC/MS analysis of the extracts also showed that the three compounds were not present in the samples. In the analysis, ions corresponding to the masses of PM130391, 130392, and 140293 were extracted to ensure that these compounds were not produced at all, Figure S19.

As mentioned before, compounds containing piperazic acid in their structure are known to have important bioactive properties [32]. The compounds isolated in this work are lipo-heptapeptides containing three piperazic acid residues that show activity against four human cancer cell lines. All of the compounds described showed a GI_50_ lower or equal to 0.1 µM, Table 3. We compared the GI_50_ values of **1**, **2**, and **3** to that of seven known antitumor compounds (5-FU, irinotecan, oxliplatin, gefinitib, erlotinib, epirubicin, and cyclophosphamide) against the same cell lines. These values were obtained from the Genomics of Drugs Sensitivity in Cancer website (Sanger data for GDSC2 dataset) [40]. Compounds **1**, **2**, and **3** showed a lower GI_50_ in all cell lines tested than all of the compounds searched (Table 3). A similar activity was that of **1** and epirubicin against lung NSCLC A549, being the GI_50_ from **1** half of that of epirubicin. Moreover, compound **3** showed a GI_50_ of 3 nM against the pancreas adenocarcinoma PSN1 cell line. According to GLOBOSCAN 2020, lung cancer is the leading cause of cancer-related deaths in the world, followed by colorectal cancer. Breast cancer has the highest incidence worldwide, although this type of cancer has less mortality than the previous two. Pancreas cancer is one of the main causes of cancer-related deaths in the world [50]. A 5-year survival rate for this cancer is about 7%, which can progress up to 22% in the case of patients suffering a resectable disease, which is, unfortunately, only in 15–20% of the cases [51]. This survival rate is significantly lower than lung, colon, and breast cancer (25%, 63%, and 90%, respectively, according to the American Cancer Society [52]). Treatment with FOLFIRINOX, which is a combination of folinic acid, 5- fluorouracil (5-FU), irinotecan, and oxaliplatin, has shown an improvement in survival rates compared to the use of gemcitabine [51]. However, this combination of chemotherapeutics is quite aggressive and cannot be applied to all patients [53]. Compound **3** has a GI_50_ at least 2000 times smaller against the pancreas cell line PSN1 than any of the compounds used in FOLFIRINOX, making it a good candidate for further studies as a possible treatment against this highly mortal cancer (Table 3).

It has been demonstrated that in these types of molecules, longer acyl chains show a higher cytotoxic activity [54]. The manipulation of the side chains in compounds 1, 2, and 3 might modulate their cytotoxicity. A better cytotoxic activity might result in lower doses required for use in patients, and therefore, hopefully, these molecules may have lower toxic effects than those currently on the market.

Further, in vivo studies, as well as stereochemistry analysis, are needed to study the potential of these compounds to be used as a treatment in the future.

## Figures and Tables

**Table 1:** Strains used in this work.

Strain Name	Details	Resistance	Reference
*Streptomyces tuirus* PHM034	Wild type strain	-	This work
*Streptomyces tuirus* PM13 Dis7 and *Streptomyces tuirus* PM13 Dis8	Disruption of the biosynthetic cluster of PM130392, PM130393 and PM140293 by insertion of the vector pOJ260 [35]	ApraR	This work
*Escherichia coli* DH5α	F-80d*lacZ* M15 *(lacZ*YA*-argF)* U169 *rec*A1 *end*A1 *hsd*R17*(*r_k_−, m_k_+) *pho*A*sup*E44λ*-thi*-1 gyrA96 *rel*A1	-	Commercial strain

**Table 2:** Primers used in this work.

Primer Name	Sequence 5′➔3′
PM13 Dis Fw	ATATTCTAGACGGGAGAGGTTGAGGAAGTC
PM13 Dis Rv	ATATGGATCCGGCTTTCGCATCGAACTC
PM13 Dis1 check Up	CGAGCTGCTCAACCTGTACG
M13 Fw	GTAAAACGACGGCCAGT
PM13 Dis1 check down	GTCGTCGAACGGGTTGTTGC
pOJ260 Check	ACACAGGAAACAGCTATGAC

**Table 3:** GI_50_ in the molar of **1**, **2** and **3** compared to that of Gefitinib, Erlotinib, 5-luorouracil (5-FU). Epirubicin, cyclophosphamide irinotecan and oxaliolatin. Data were obtained from the Genomics of Drugs Sensitivity in Cancer website [40].

Compounds	Breast	Colon	Lung	Pancreas
	MDA-MB-231	HT29	NSCLC A549	PSN1
PM130391 (**1**)	4.60 × 10^−8^	4.60 × 10^−8^	1.00 × 10^−7^	1.90 × 10^−8^
PM130392 (**2**)	1.60 × 10^−8^	2.00 × 10^−8^	4.80 × 10^−8^	7.00 × 10^−9^
PM140293 (**3**)	1.60 × 10^−8^	2.10 × 10^−8^	3.20 × 10^−8^	3.00 × 10^−9^
Gefitinib	3.48 × 10^−5^	1.74 × 10^−4^	1.33 × 10^−5^	5.18 × 10^−5^
Erlotinib	1.99 × 10^−5^	6.48 × 10^−5^	8.12 × 10^−6^	2.35 × 10^−5^
5-FU	6.67 × 10^−5^	2.48 × 10^−5^	9.96 × 10^−6^	6.41 × 10^−6^
Epirubicin	1.50 × 10^−7^	5.90 × 10^−7^	2.10 × 10^−7^	1.10 × 10^−7^
Cyclophosphamide	1.96 × 10^−4^	6.64 × 10^−4^	3.29 × 10^−4^	1.32 × 10^−4^
Irinotecan	1.55 × 10^−5^	4.52 × 10^−5^	1.30 × 10^−5^	7.71 × 10^−6^
Oxaliplatin	3.73 × 10^−5^	1.16 × 10^−5^	4.98 × 10^−6^	2.23 × 10^−5^

**Table 4:** ^1^H and ^13^C NMR data of Compound 1 in CDCl_3_.

Unit Position	*δ*_C_	*δ*_H_ (*J* in Hz)	Unit Position	*δ*_C_	*δ*_H_ (*J* in Hz)
Ala			DHPA		
1-C	171.9		1-C	171.1	
2-CH	48.2	4.10 (d, 6.7)	2-CH	50.7	5.28 (d, 6.6, 1.7)
3-CH_3_	19.9	1.18 (d, 6.8)	3-CH_2_	25.6	2.48 (ddt, 13.0, 6.2, 1.8)
NH		6.68 (d, 6.4)			1.98 (m)
Thr			4-CH	59.6	4.57 (dd, 12.0, 6.2)
1-C	171.3		5-CH	149.8	7.01 (s)
2-CH	49.5	5.95 (dd, 7.1, 3.2)			
3-CH	69.4	5.19 (dd, 6.5, 3.4)	N-Me-Gly		
4-CH_3_	15.7	1.30 (d, 6.5)	1-C	168.9	
NH		6.99 (d, 7.1)	2-CH_2_	51.3	4.97 (d, 17.5)
PA-1					3.53 (d, 17.5)
1-C	172.9		N-CH_3_	36.8	3.19 (s)
2-CH	49.2	5.64 (dd, 6.5, 1.8)	N-OH-Ala		
3-CH_2_	25.3	1.90 (m)	1-C	169.7	
		2.07 (m)	2-CH	53.3	5.20 (t, 7.4)
4-CH_2_	21.5	1.56 (m)	3-CH_3_	14.3	1.51 (d, 7.4)
5-CH_2_	47.3	3.05 (d, 13.5)	2,8 DMNA		
		2.67 (m)	1-C	175.7	
NH		5.4 (dd, 13.2, 1.9)	2-CH	41.3	2.17 (m)
PA-2			3-CH_2_	34.4	1.59 (m)
1-C	172.7				1.33 (m)
2-CH	43.8	6.09 (dd 6.6, 2.0)	4-CH_2_	27.4	1.24 (m)
3-CH_2_	23.5	1.97 (m)	5-CH_2_	29.8	1.24 (m)
4-CH_2_	19.6	2.14 (m)	6-CH_2_	27.2	1.24 (m)
		1.54 (m)	7-CH_2_	38.9	1.23 (m)
5-CH_2_	47.4	3.16 (m)			1.13 (m)
		2.75 (dt, 13.3, 3.4)	8-CH	27.9	1.50 (m)
NH		4.80 (dd, 12.9, 1.8)	9-CH_3_	22.6	0.86 (d, 6.6)
			10-CH_3_	22.6	0.86 (d, 6.6)
			11-CH_3_	17.5	1.10 (d, 6.8)

## Data Availability

The data presented in this study are available on request from the corresponding author. The data are not publicly available due privacy.

## Supplementary Materials

The following supporting information can be downloaded at: https://www.mdpi.com/article/10.3390/metabo13101091/s1, Figure S1: ^1^H NMR (500 MHz) Spectrum of Compound 1 in CDCl_3_, Figure S2: ^13^C NMR (100 MHz) Spectrum of Compound 1 in CDCl_3_, Figure S3:_:_ gCOSY (500 MHz) Spectrum Compound 1 in CDCl_3_, Figure S4: TOCSY (500 MHz) Spectrum of Compound 1 in CDCl_3_, Figure S5: gHSQC (500 MHz) Spectrum of Compound 1 in CDCl_3_, Figure S6: gHMBC (500 MHz) Spectrum of Compound 1 in CDCl_3_, Figure S7: ^1^H NMR (500 MHz) Spectrum of Compound 2 (mixture of 2a 58% and 2b 42%) in CDCl_3_, Figure S8: ^13^C NMR (100 MHz) Spectrum of Compound 2 (mixture of 2a 58% and 2b 42%) in CDCl_3_, Figure S9: ^13^C NMR (100 MHz) Spectrum of Compound 2 (mixture of 2a 58% and 2b 42%) in CDCl_3_, Figure S10: gCOSY (500 MHz) Spectrum Compound 2 (mixture of 2a 58% and 2b 42%) in CDCl_3_, Figure S11: gHSQC (500 MHz) Spectrum of Compound 2 (mixture of 2a 58% and 2b 42%) in CDCl_3_, Figure S12: gHMBC (500 MHz) Spectrum of Compound 2 (mixture of 2a 58% and 2b 42%) in CDCl_3_, Figure S13: ^1^H NMR (400 MHz) Spectrum of Compound 3 in CDCl_3_, Figure S14: ^13^C NMR (100 MHz) Spectrum of Compound 3 in CDCl_3_, Figure S15: HRESIMS *m*/*z* 849.4853 (calcd for C_39_H_65_N_10_O_11_, 849.4829) of compound 1, Figure S16: HRESIMS *m*/*z* 863.5055 (calcd for C_40_H_67_N_10_O_11_, 863.4985) of compound 2, Figure S17: HRESIMS *m*/*z* 877.5192 (calcd for C_41_H_69_N_10_O_11_, 877.5142) of compound 3, Figure S18: PCR check of the mutant strains, Figure S19: HPLC-MS results from the extract of the wild type strain and *S. tuirus* PM13 Dis7, Table S1: Clusters predicted by antiSMASH and PRISM in the genome of *S. tuirus* PHM034 [38,55]. Table S2: Genes predicted in PM130391, 130392, and 140293 biosynthetic gene cluster and predicted function of the encoded enzymes. Table S3. Blastp results of every predicted enzyme encoded in the gene cluster.
