# Supplementary material for: Identification of the Biosynthetic Gene Cluster of New Piperazic Acid-Containing Lipopeptides with Cytotoxic Activity in the Genome of Marine Streptomyces PHM034
Source: Metabolites. 2023 Oct 18;13(10):1091. doi: 10.3390/metabo13101091 (PMC10609185; doi:10.3390/metabo13101091)
Supplement: Supplementary file 1 [file metabolites-13-01091-s001.zip › Supplementary.pdf]

Identification of the Biosynthetic Gene Cluster of New Piperazic Acid-Containing Lipopeptides with Cytotoxic Activity in the Genome of Marine Streptomyces PHM034

Ana Ceniceros [1,2], Librada Cañedo [3], Carmen Méndez [1,2], Carlos Olano [1,2], Carmen Schleissner [4], Carmen Cuevas [3], Fernando de la Calle [3] and José A. Salas [1,2,*]

[1] Departamento de Biología Funcional e Instituto Universitario de Oncología del Principado de Asturias (IUOPA), Universidad de Oviedo, 33006 Oviedo, Spain; acmedrano@alumni.unav.es (A.C.); cmendezf@uniovi.es (C.M.); olanocarlos@uniovi.es (C.O.)
[2] Instituto de Investigación Sanitaria del Principado de Asturias, (ISPA), 33006 Oviedo, Spain
[3] Drug Discovery Area, PharmaMar S.A. Avda. de los Reyes 1, Colmenar Viejo, 28770 Madrid, Spain; lcanedo@pharmamar.com (L.C.); ccuevas@pharmamar.com (C.C.); fdelacalle@pharmamar.com (F.d.l.C.)
[4] Unolab Manufacturing, Avenida de las Flores 6, Humanes de Madrid, 28970 Madrid, Spain; cschleissner@unolab.es
* Correspondence: jasalas@uniovi.es

Table of contents **Pages**

Figures S1-S6. 1D and 2D NMR spectra of **1** in $CDCl_3$. 2-7
Figures S7-S12. 1D and 2D NMR spectra of **2** in $CDCl_3$. 8-13
Figures S13-S14. 1D spectra of **3** in $CDCl_3$. 14-15
Figures S15-S17. HRESIMS of **1**-**3**. 16-18
Figure S18 PCR check of the mutant strains. 19
Figure S19 HPLC-MS results from the extract of the wild type strain and *S. tuirus* PM13 Dis7 20
Table S1 Clusters predicted by antiSMASH in the genome of *S. tuirus* PHM034 21
Table S2. Genes predicted in PM130391, 130392, and 140293 biosynthet gene cluster and predicted function of the encoded enzymes. 22-23

Figure S1

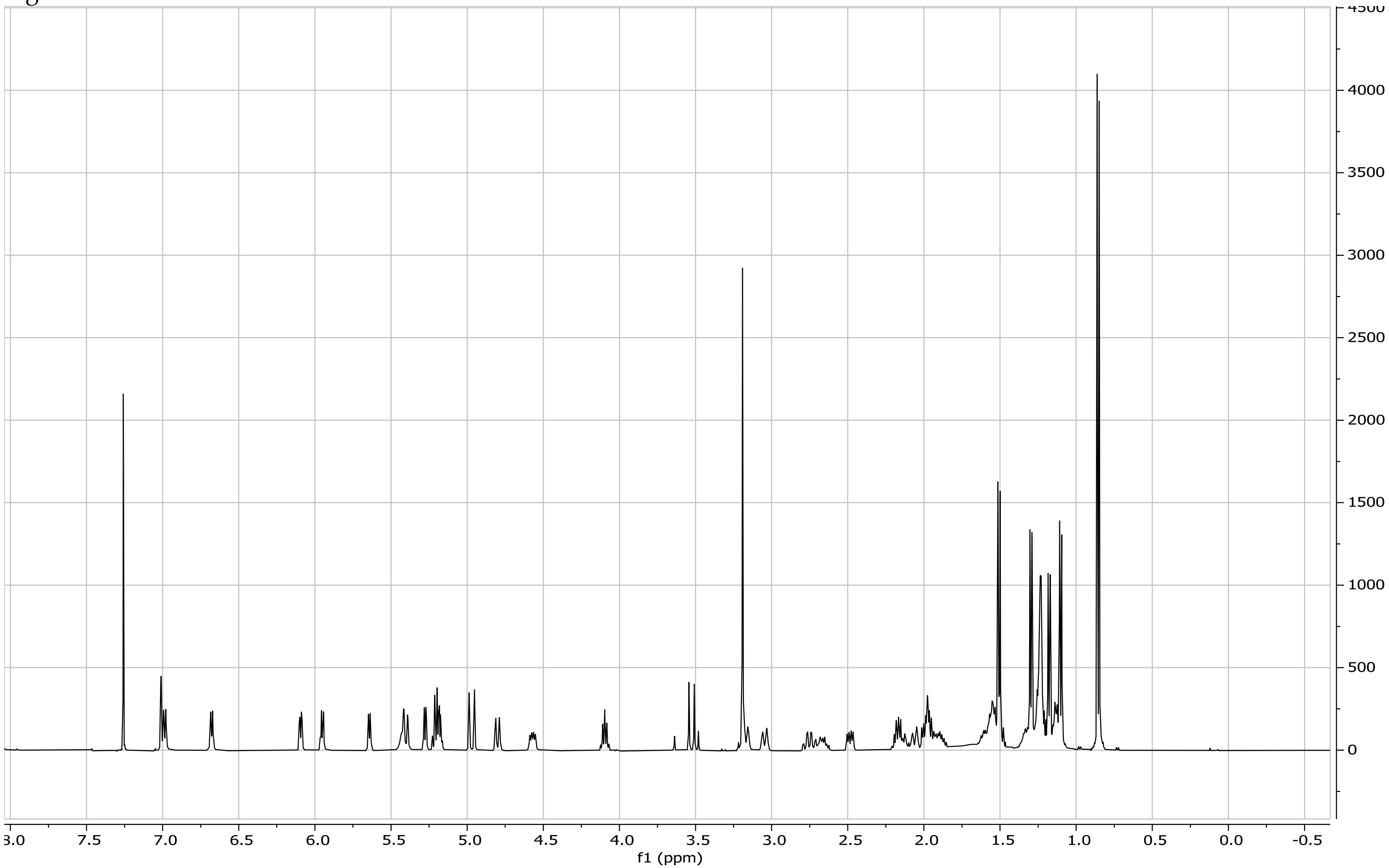

Figure S1. ^{1}H NMR (500 MHz) Spectrum of Compound **1** in $CDCl_3$

Figure S2

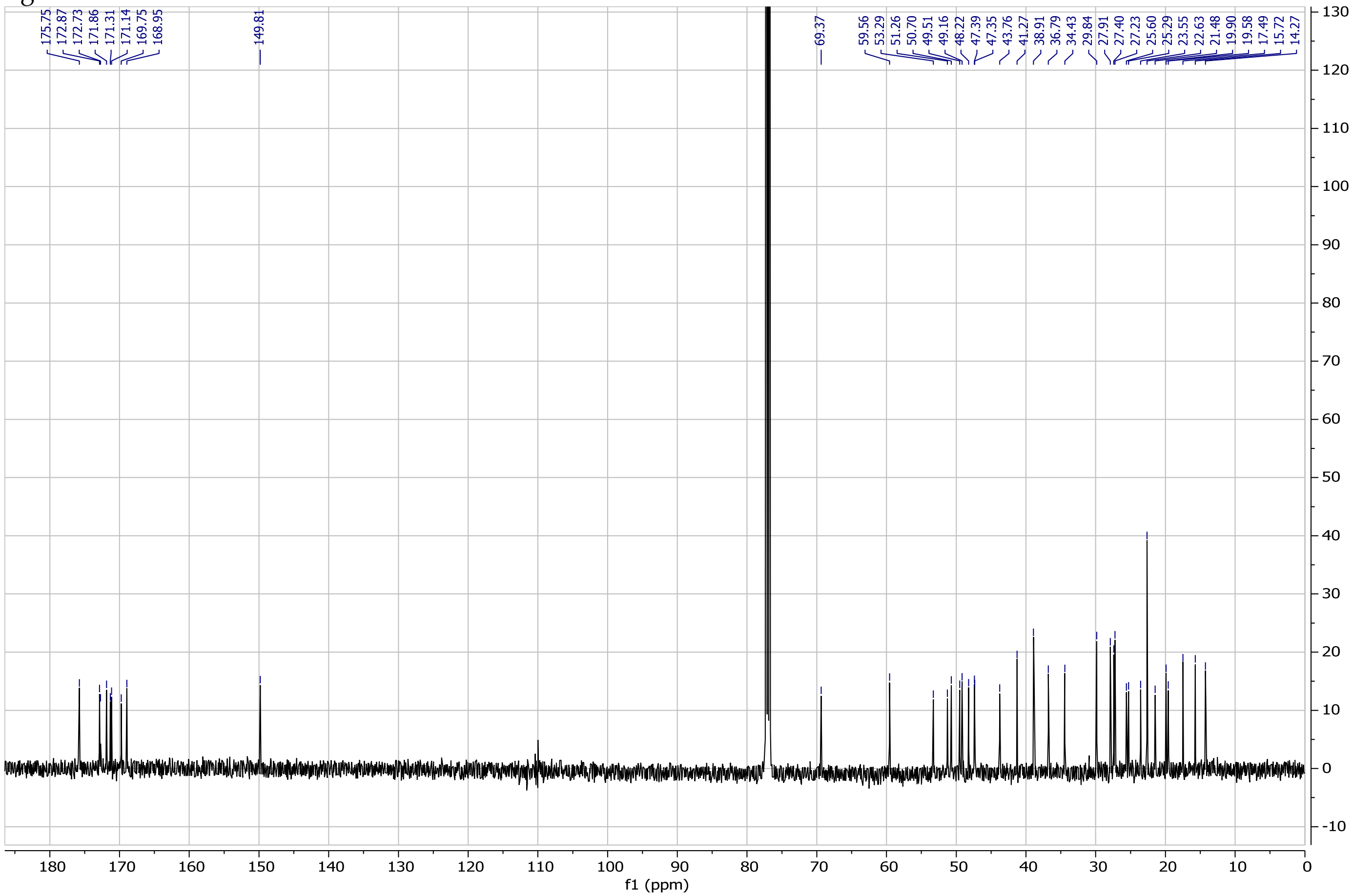

Figure S2. [13]**C** NMR (100 MHz) Spectrum of Compound **1** in $CDCl_3$

Figure S3

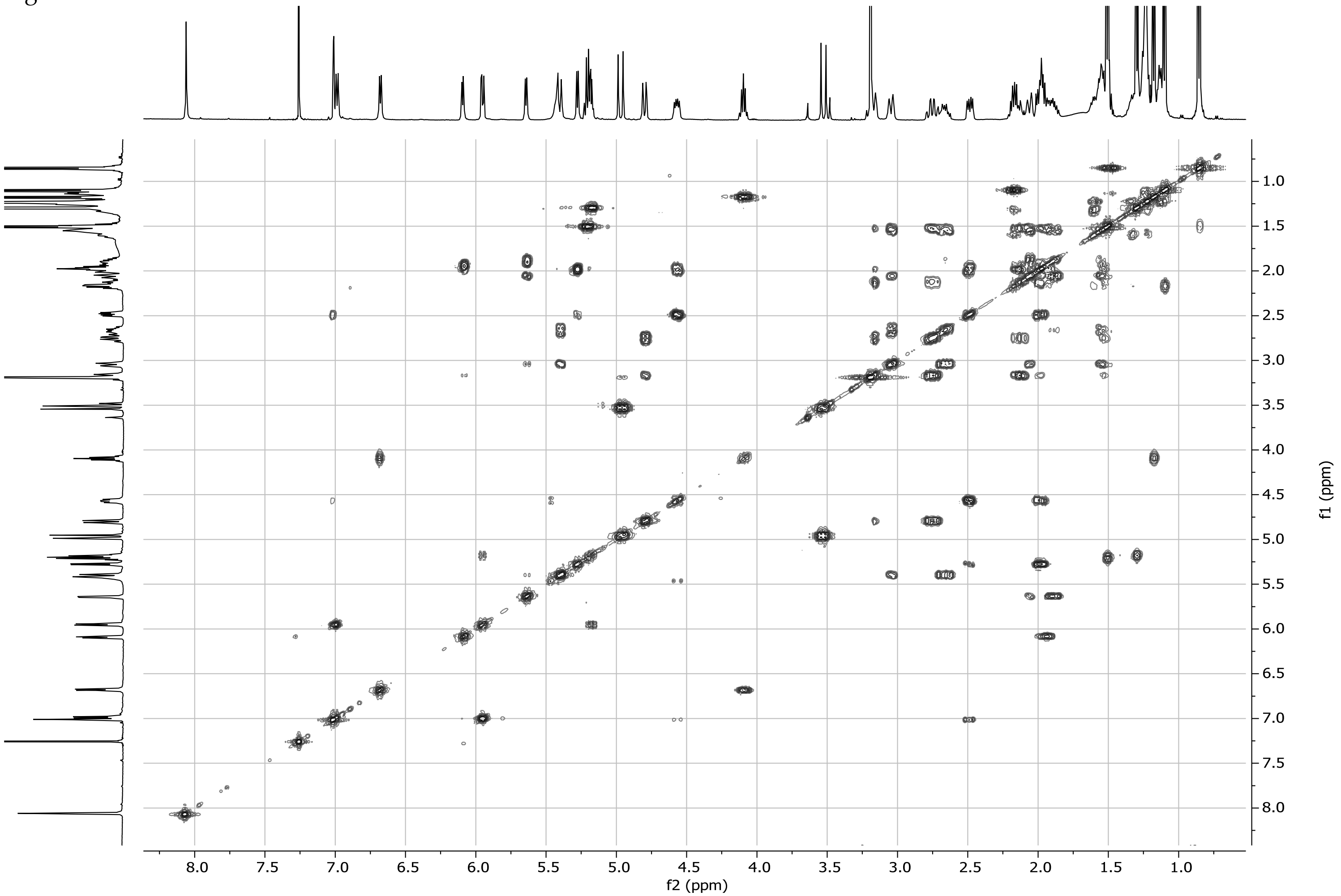

Figure S3. gCOSY (500 MHz) Spectrum Compound **1** in $CDCl_3$

Figure S4

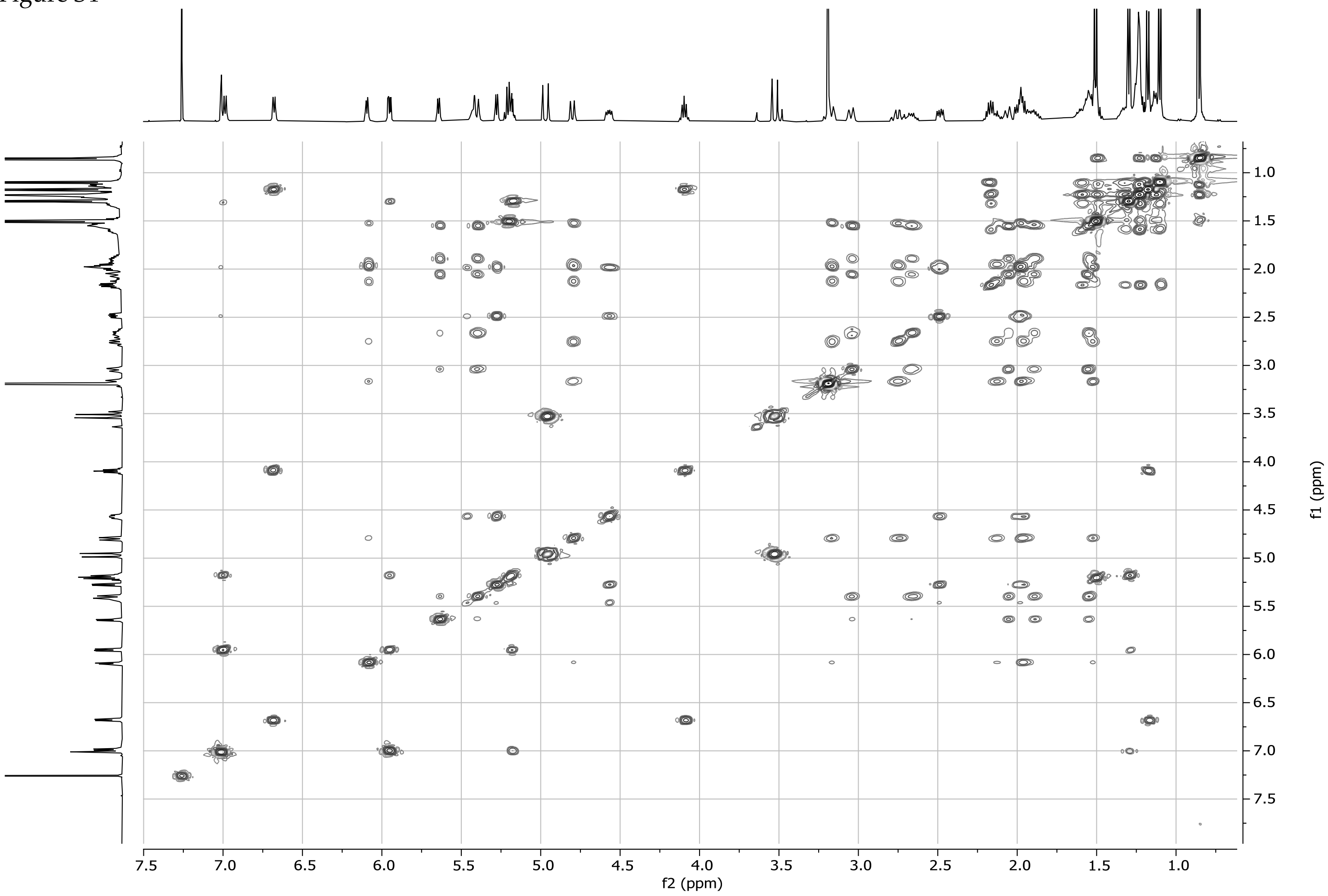

Figure S4. TOCSY (500 MHz) Spectrum of Compound **1** in $CDCl_3$

Figure S5

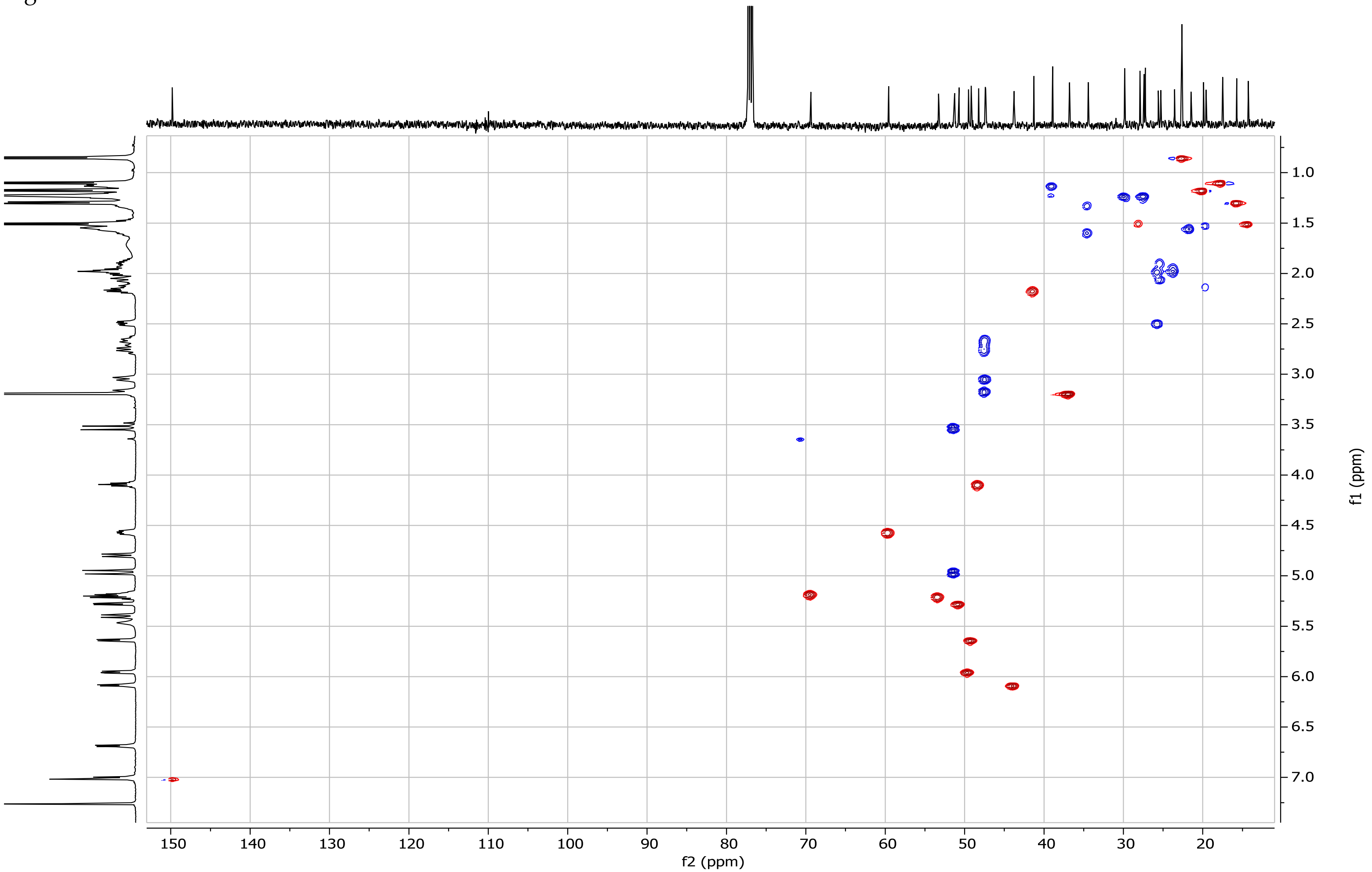

Figure S5. gHSQC (500 MHz) Spectrum of Compound **1** in $CDCl_3$

Figure S6

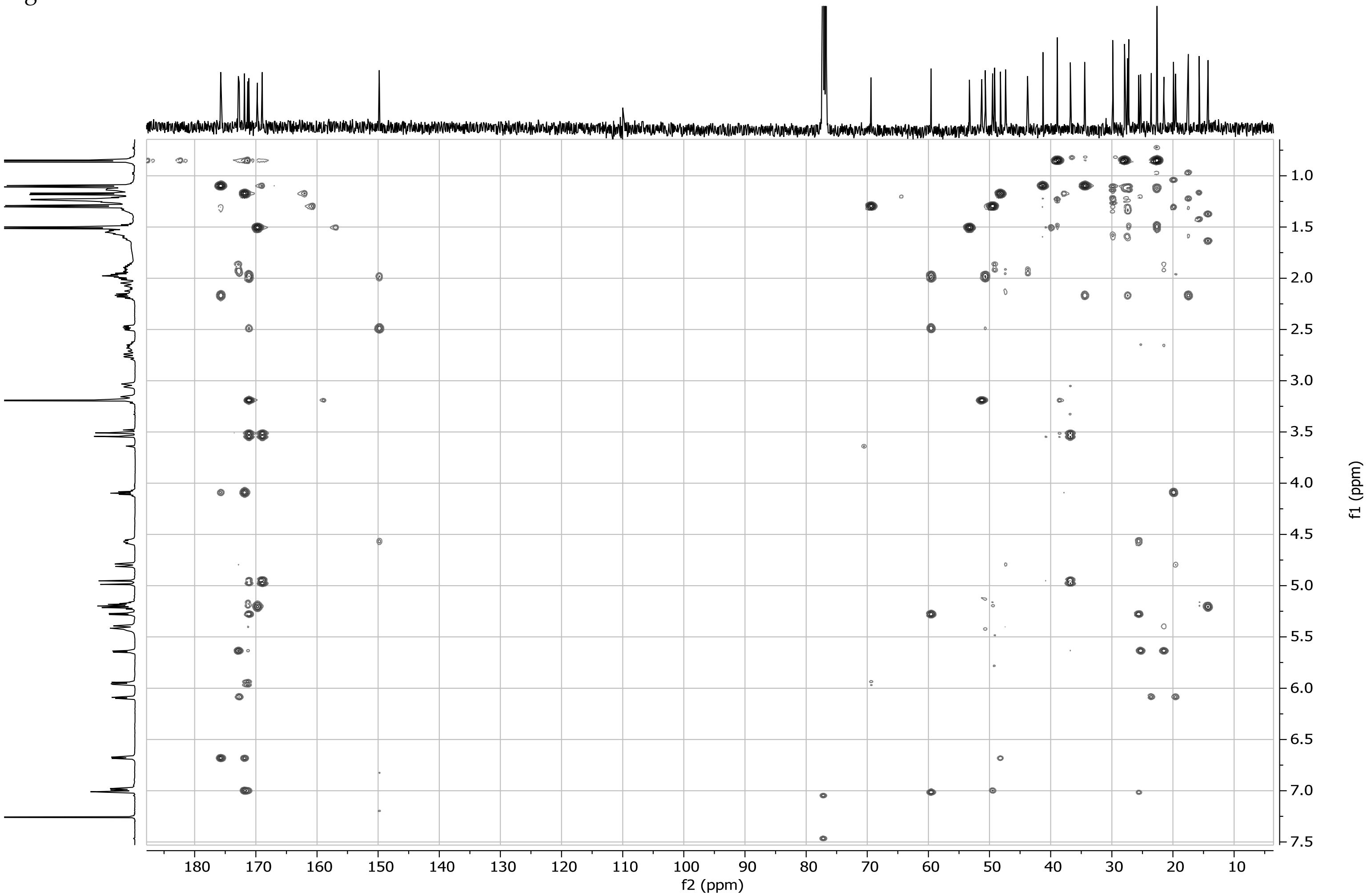

Figure S6. gHMBC (500 MHz) Spectrum of Compound **1** in $CDCl_3$

Figure S7

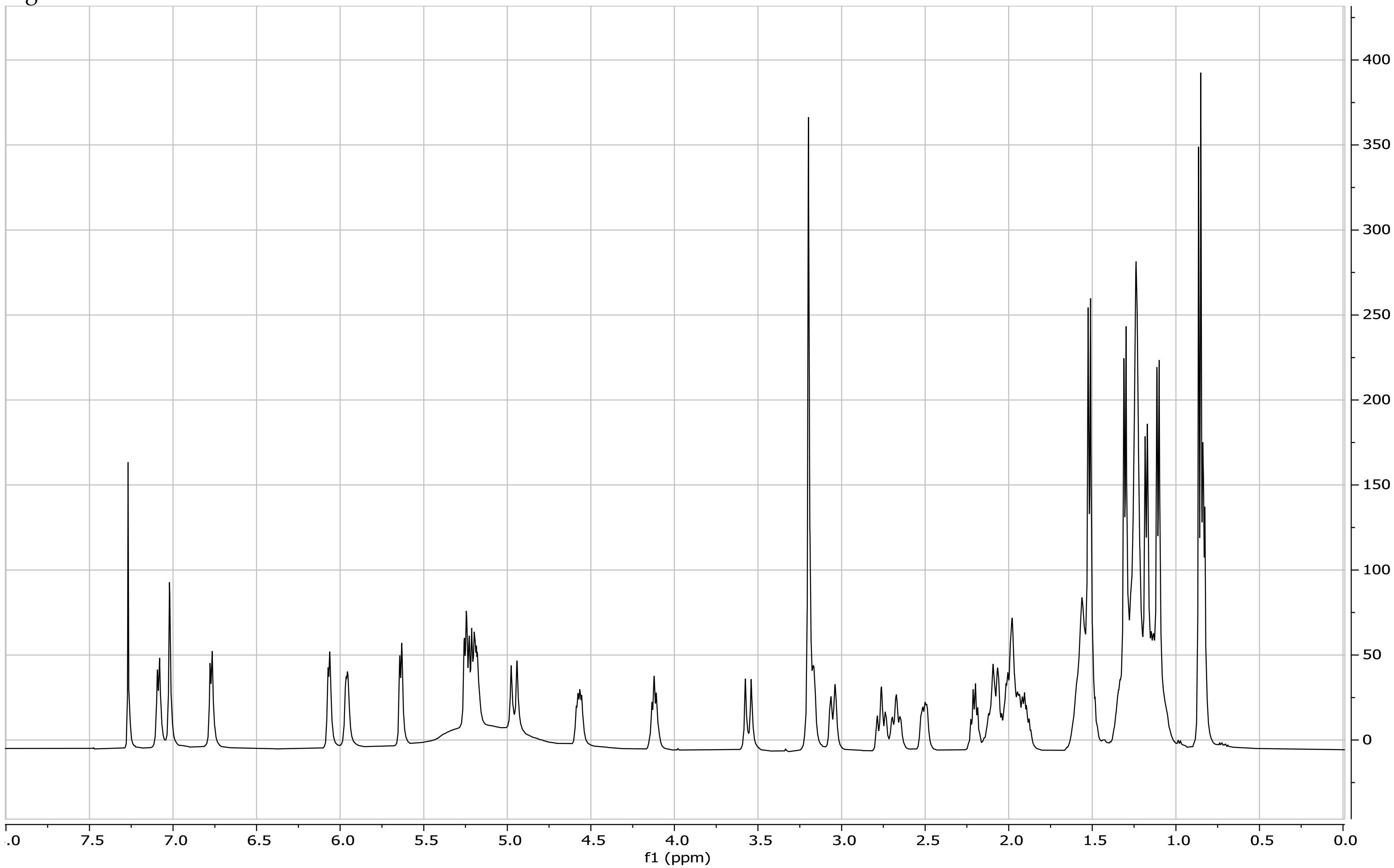

Figure S7. ^{1}H NMR (500 MHz) Spectrum of Compound **2 (mixture of 2a 58% and 2b 42%)** in $CDCl_3$

Figure S8

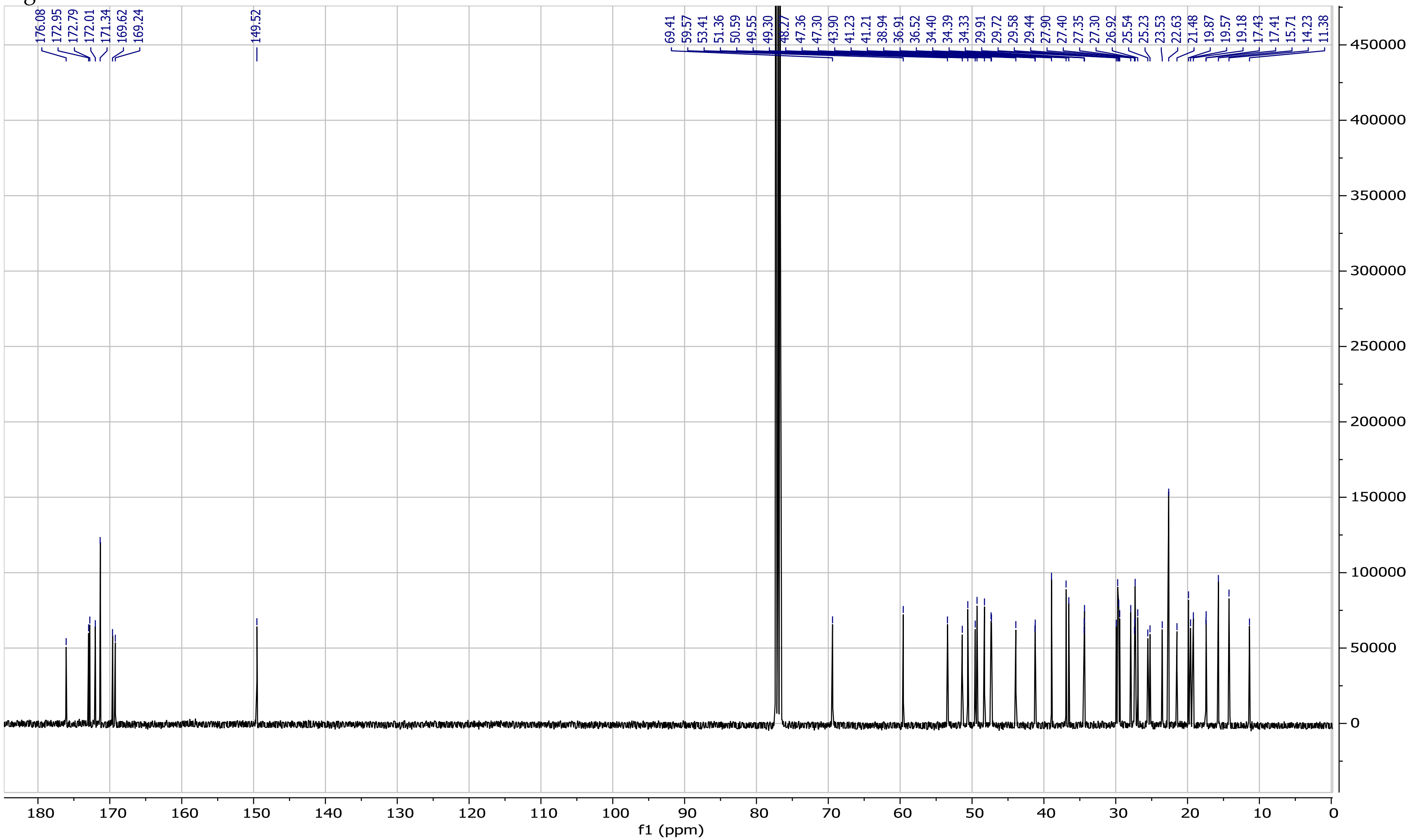

Figure S8. ^{13}**C** NMR (100 MHz) Spectrum of Compound **2 (mixture of 2a 58% and 2b 42%)** in $CDCl_3$

Figure S9

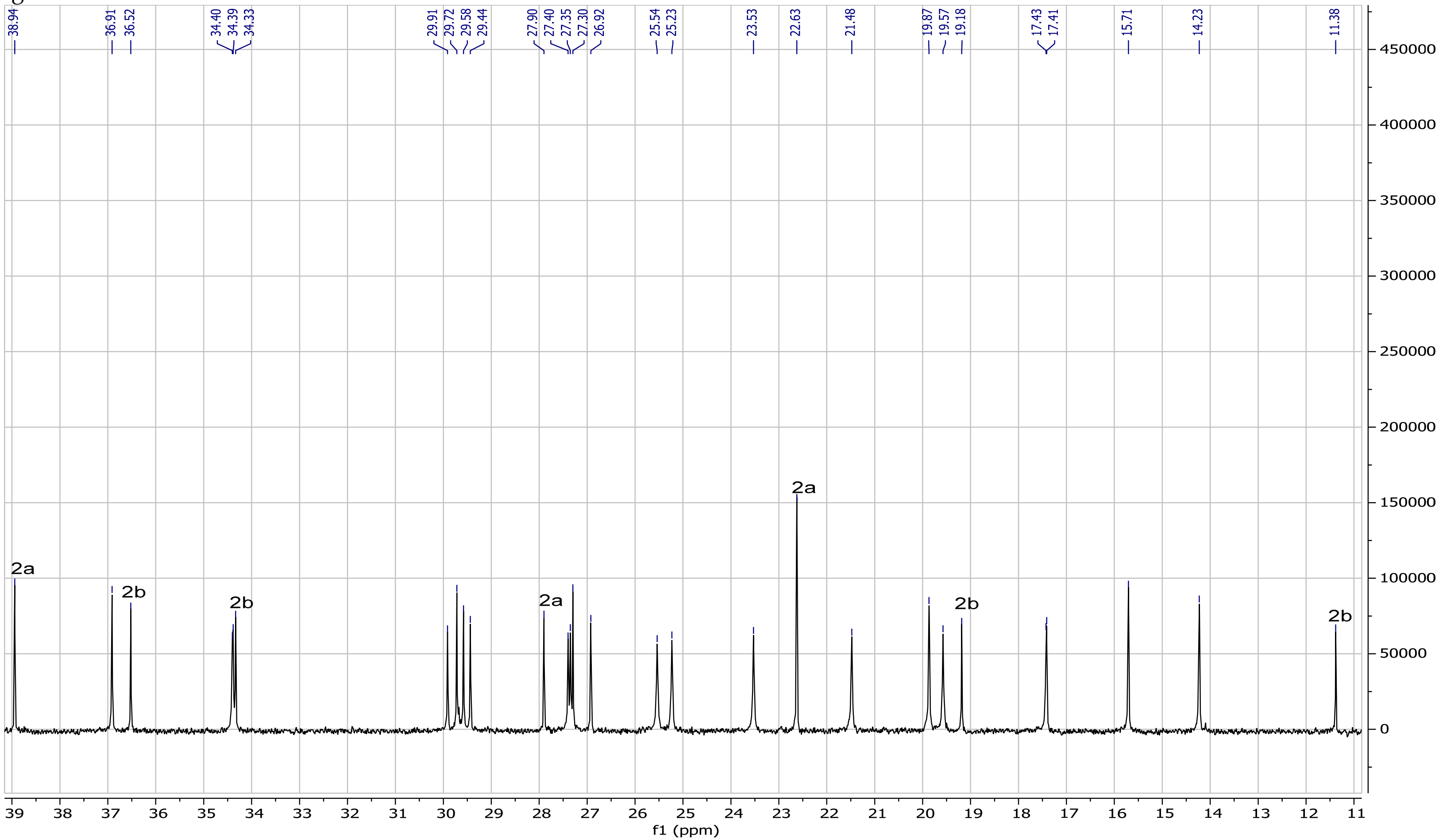

Figure S9.[13]**C** NMR (100 MHz) Spectrum of Compound **2 (mixture of 2a 58% and 2b 42%)** in $CDCl_3$

Figure S10

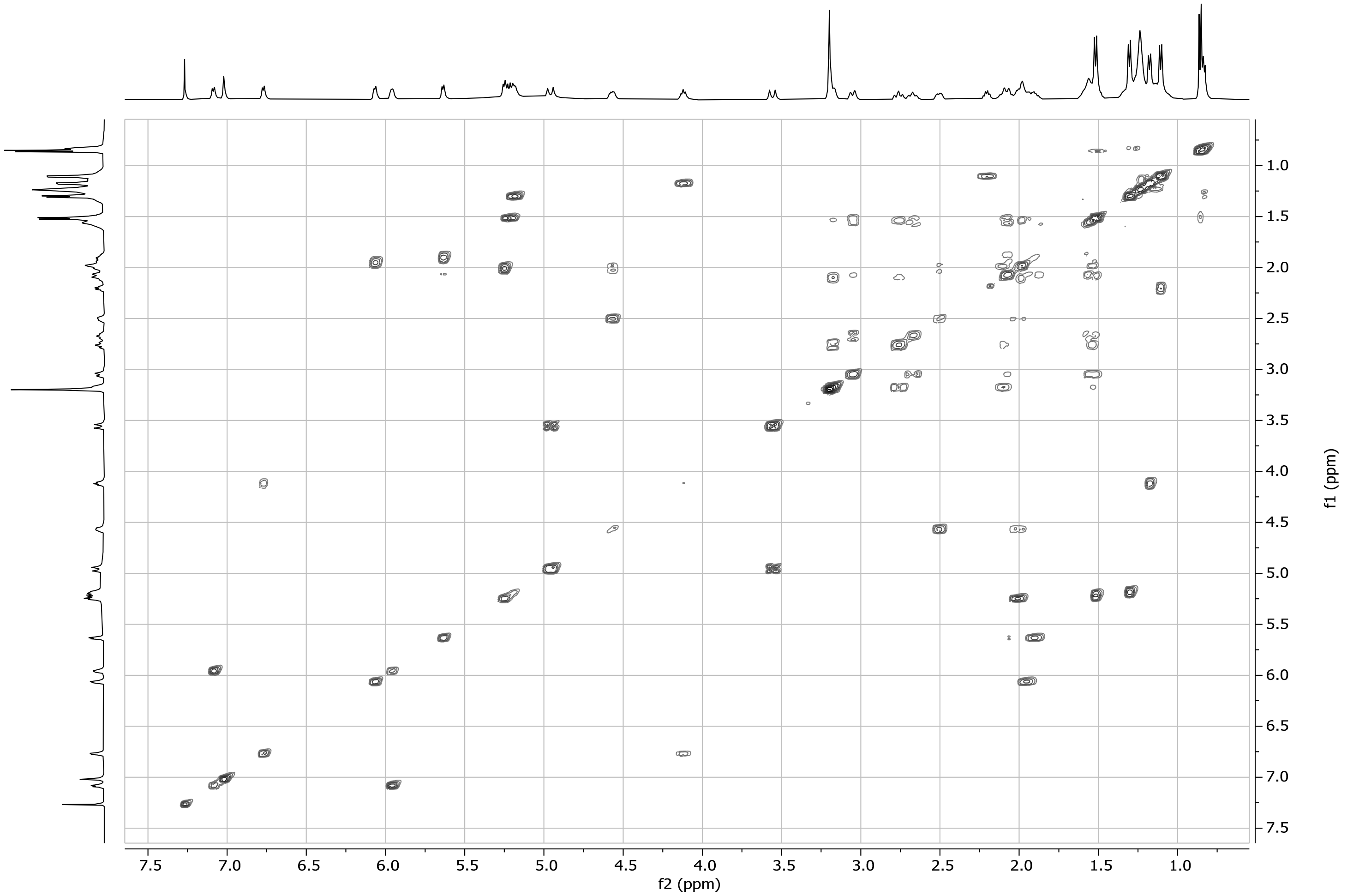

Figure S10. gCOSY (500 MHz) Spectrum Compound **2 (mixture of 2a 58% and 2b 42%)** in $CDCl_3$

Figure S11

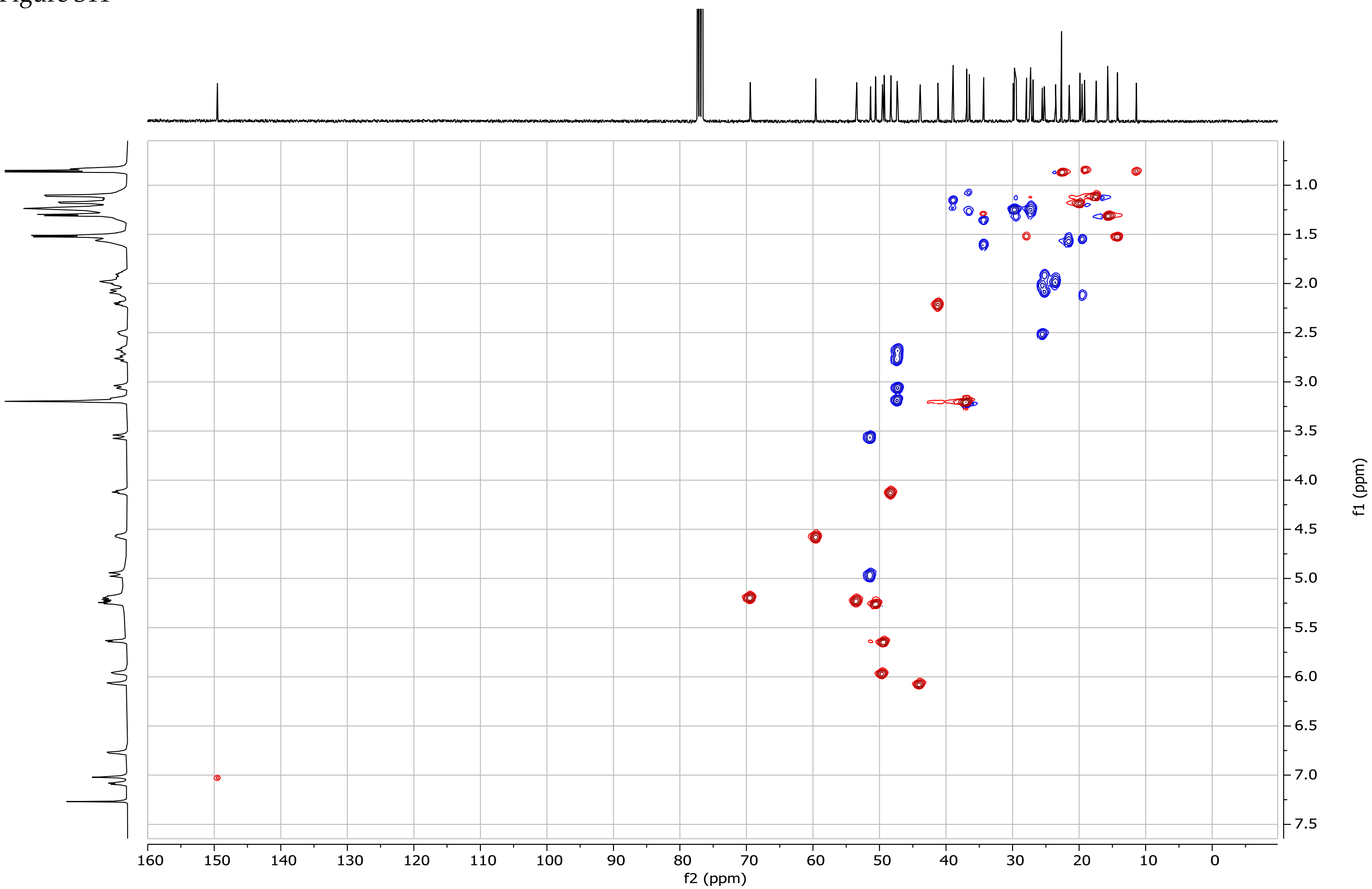

Figure S11. gHSQC (500 MHz) Spectrum of Compound **2 (mixture of 2a 58% and 2b 42%)** in $CDCl_3$

Figure S12

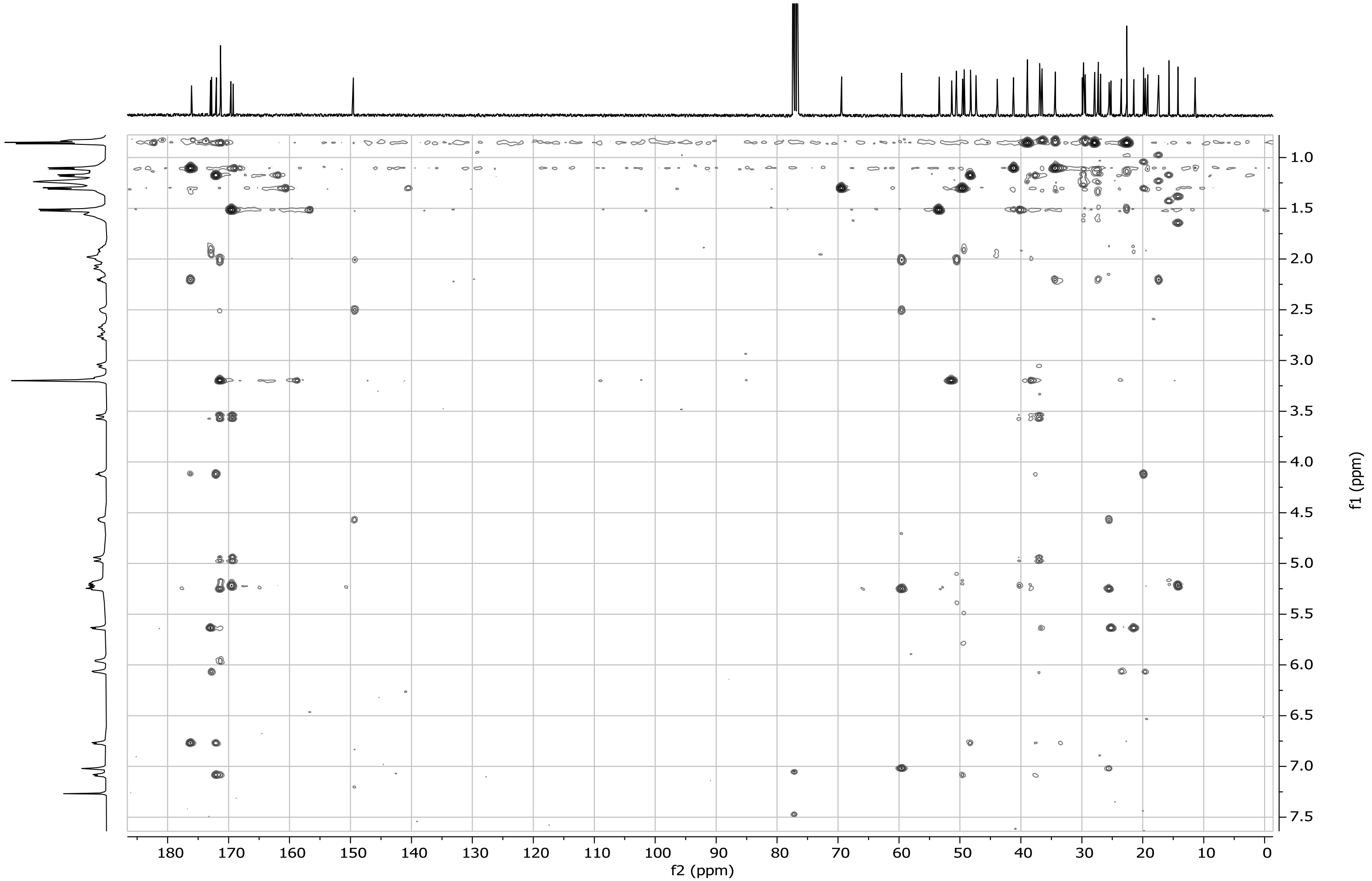

Figure S12. gHMBC (500 MHz) Spectrum of Compound **2 (mixture of 2a 58% and 2b 42%)** in $CDCl_3$

Figure S13

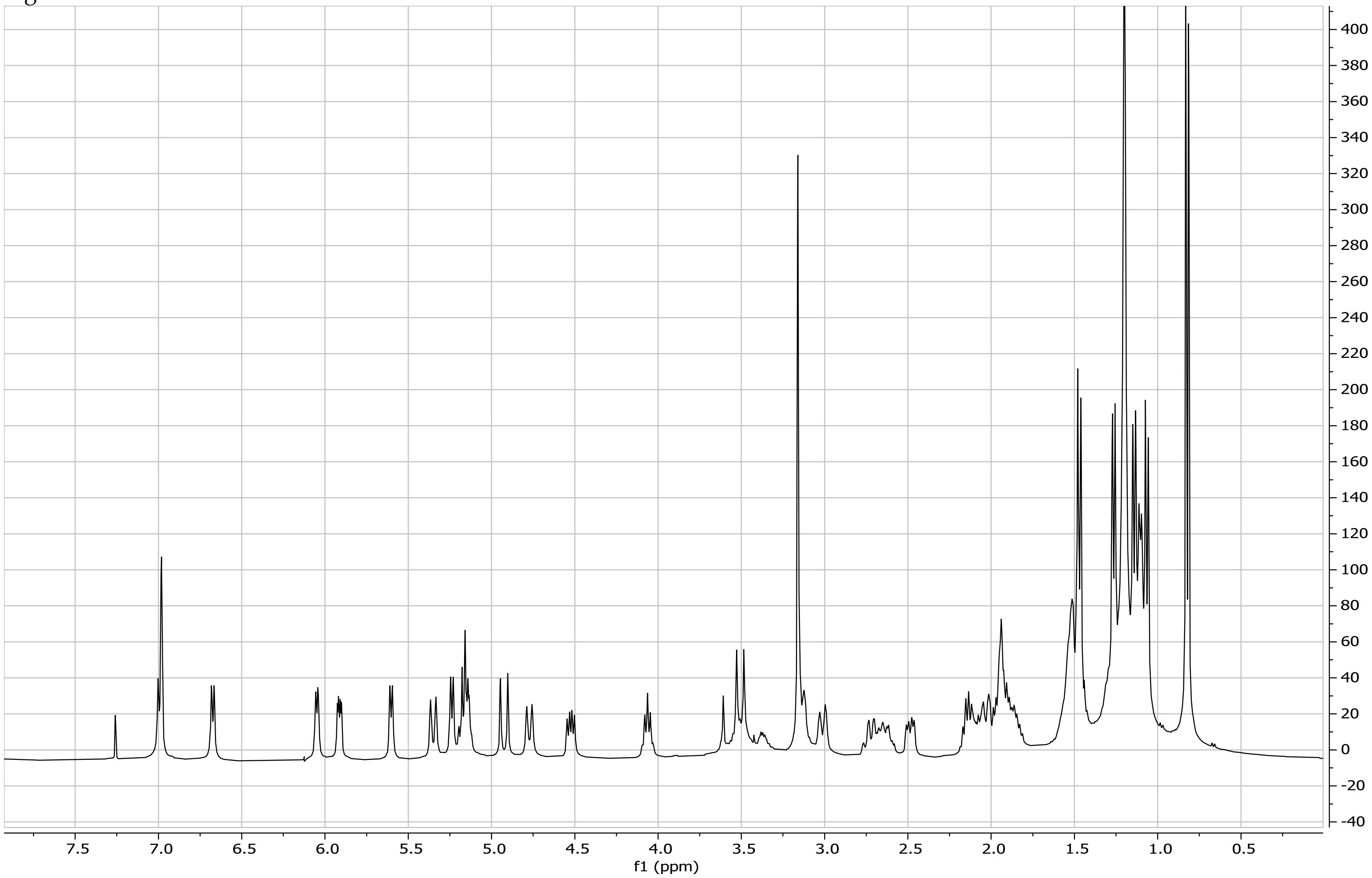

Figure S13. ^{1}H NMR (400 MHz) Spectrum of Compound **3** in $CDCl_3$

Figure S14

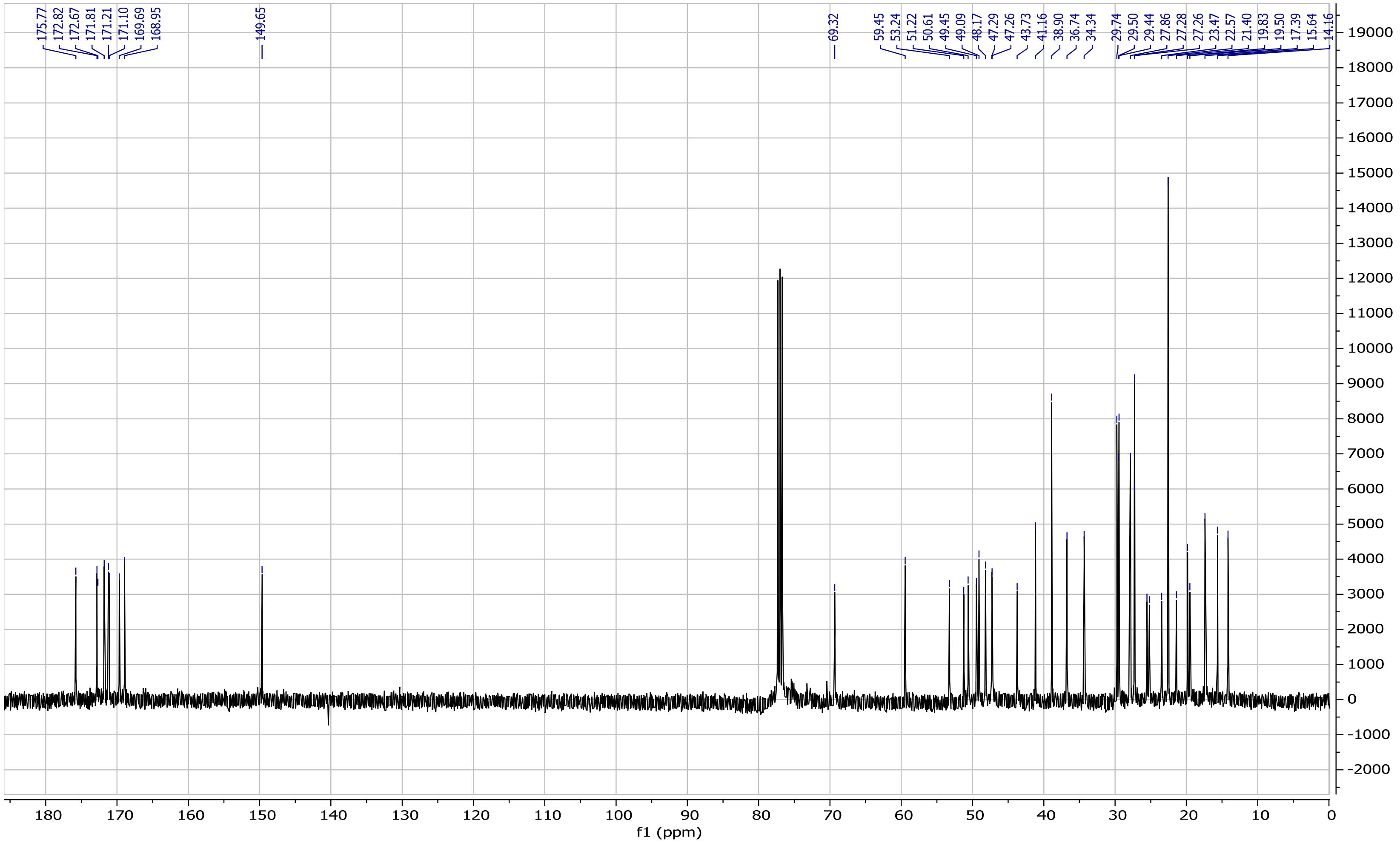

Figure S14.[13]**C** NMR (100 MHz) Spectrum of Compound **3** in $CDCl_3$

Figure S15

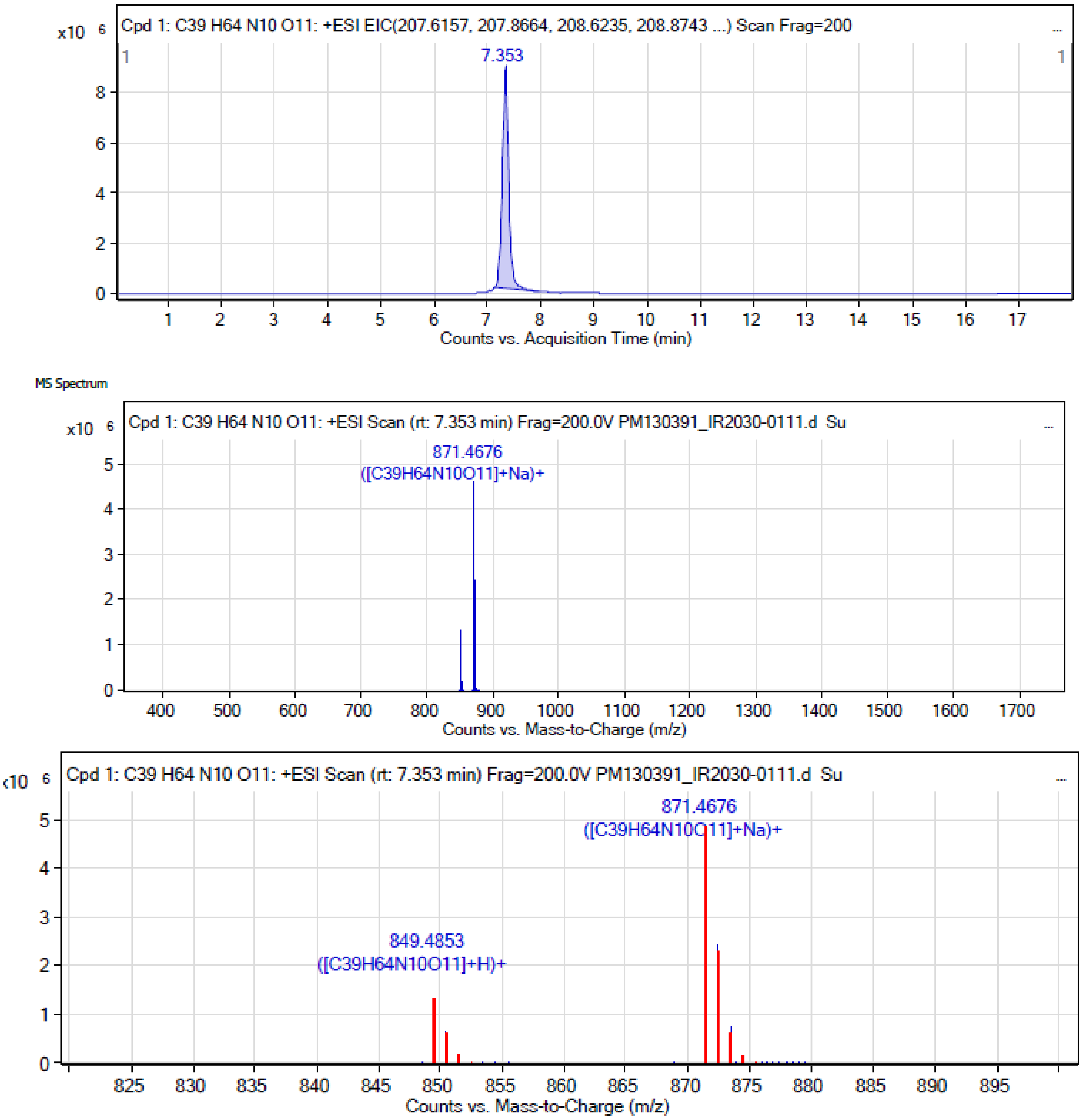

Figure S15. HRESIMS m/z 849.4853 (calcd for $C_{39}H_{65}N_{10}O_{11}$, 849.4829) of compound **1**.

Figure S16

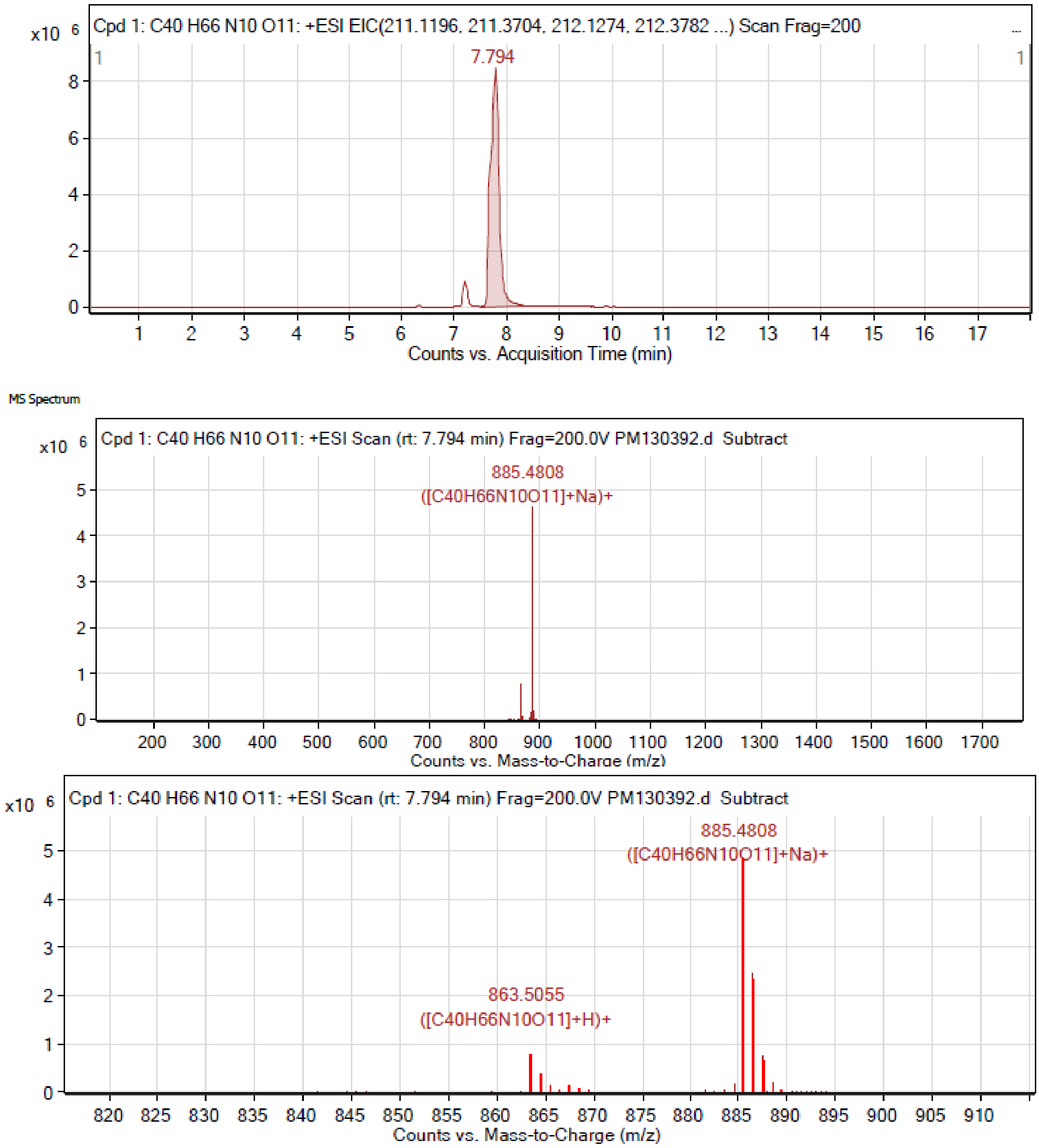

Figure S16. HRESIMS m/z 863.5055 (calcd for $C_{40}H_{67}N_{10}O_{11}$, 863.4985) of compound **2.**

Figure S17

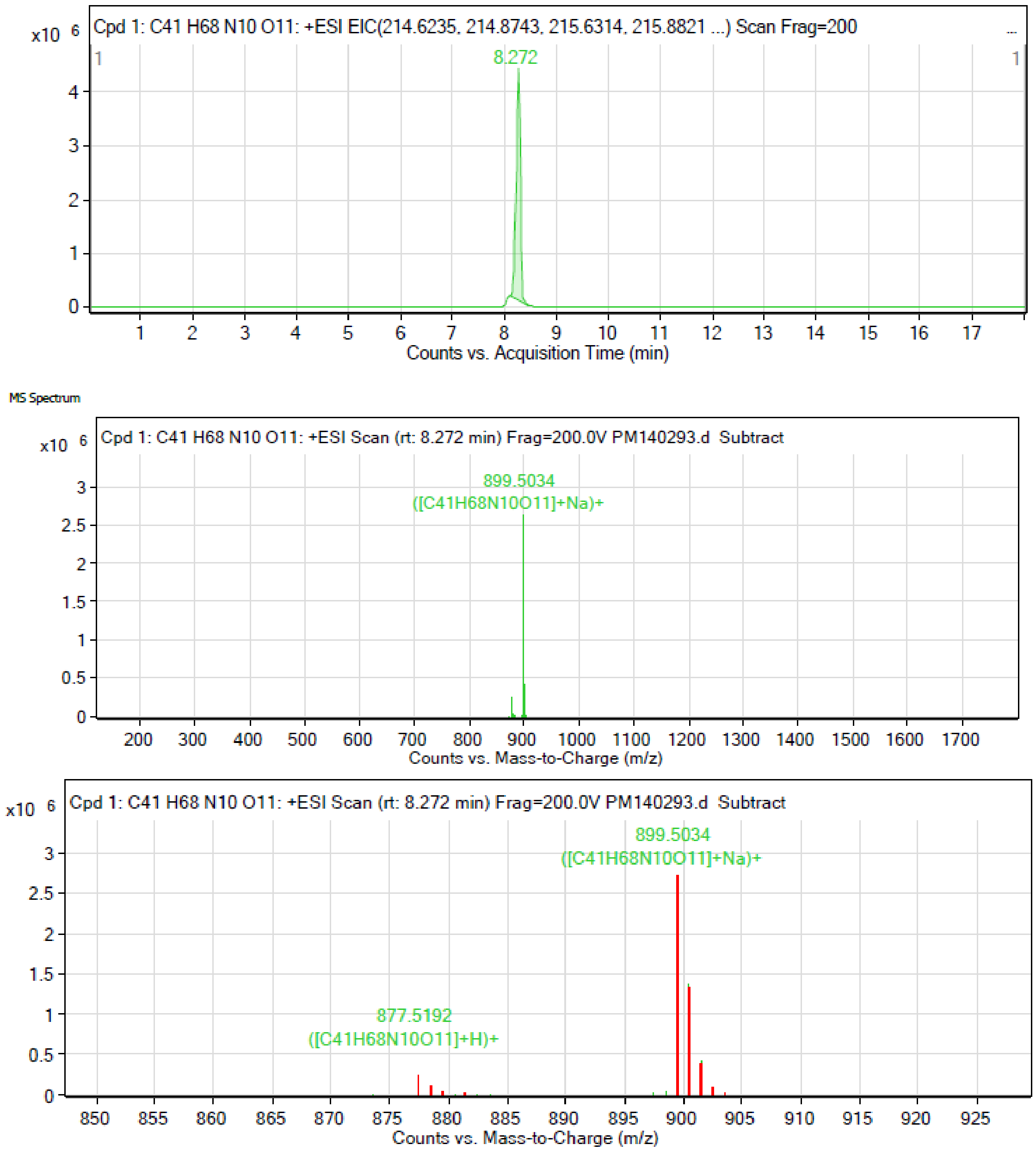

Figure S17. HRESIMS m/z 877.5192 (calcd for $C_{41}H_{69}N_{10}O_{11}$, 877.5142) of compound **3**.

Figure S18

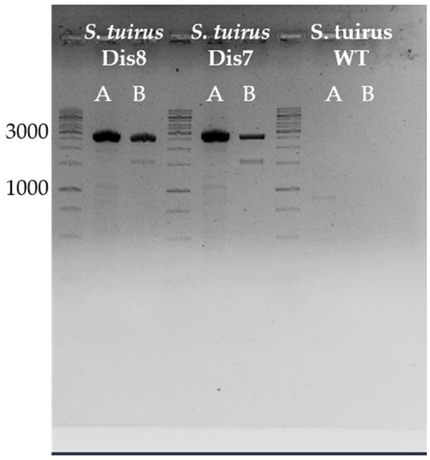

Figure S18. PCR check of the mutant strains. PCR was performed using primers annealing in the vector pOJ260 and in the chromosome outside the homologous region. The WT strain did not show any bands since primer pOJ260 check and M13 Fw do not anneal in the chromosome. A: PCR product obtained with primer PM13Dis down and pOJ260 check (2869 kb) B: PCR product obtained using primers PM13Dis Check-up and M13 Fw (2681). Primers sequence is listed in table 4).

Figure S19

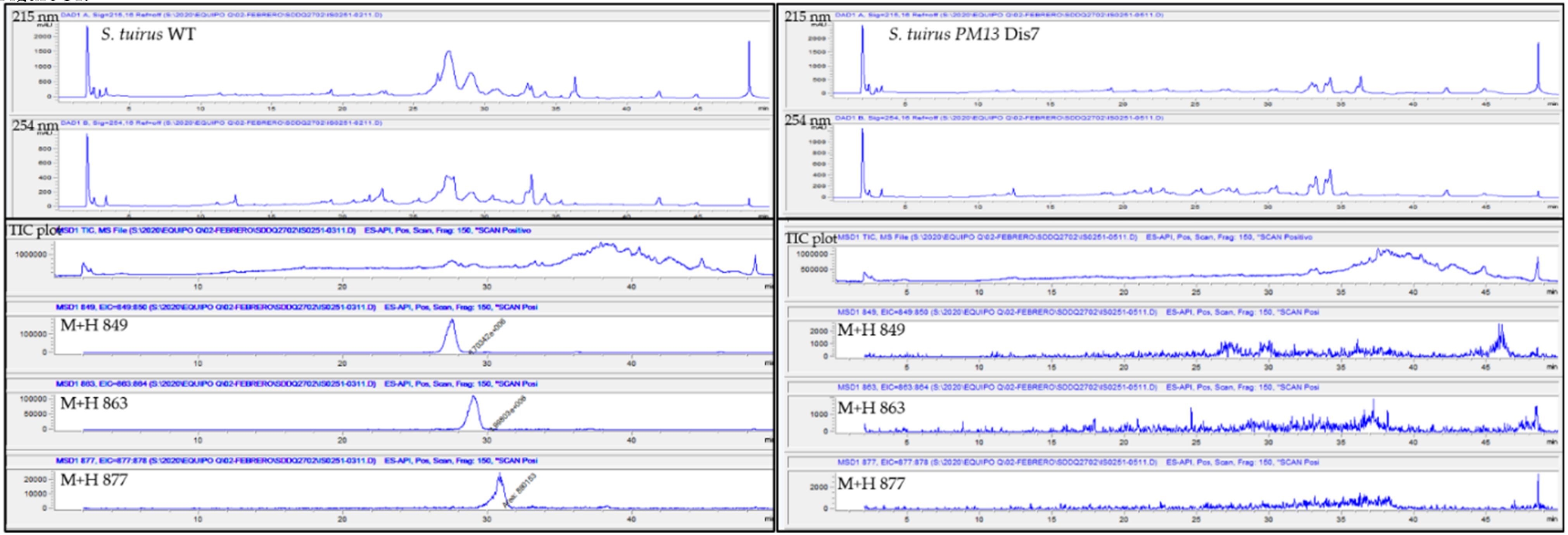

Figure S19 HPLC-MS results from the extract of the wild type strain and *S. tuirus* PM13 Dis7. The peaks corresponding to compounds 1-3 are visible in the wild type chromatogram at both 215 nm and 254 nm. However these peaks cannot be detected in the extract of *S. tuirus* PM13 Dis7 strain. When extracting the ions of the mases of the three compounds (M+H 849, 863 and 877), the thee of them can be observed in *S. tuirus* wild type strain but they are not detected in *S. tuirus* PM13 Dis7 strain.

Table S1

Table S1. BGCs predicted by antiSMASH. Similarity to known clusters is given by antiSMASH comparing each cluster with the MiBIG database [38,55].

Cluster	Type of BGC	Most similar cluster predicted by antiSMASH		Similarity
		**Known compound**	**Type of BGC**	
Contig 1 (GenBank accesion numbre JAGTPG010000002)				
Cluster 1	terpene	phenalamide A2	NRP + Polyketide	50%
Cluster 2	melanin	melanin	Other	42%
Cluster 3	NRPS,T3PKS	coelichelin	NRP	72%
Cluster 4	NRPS-like	Vazabitide A	NRP	6%
Cluster 5	ectoine	ectoine	Other	100%
Cluster 6	T1PKS	rifamycin	Polyketide	7%
Cluster 7	T1PKS, lassopeptide	Chaxapeptin	RiPP	42%
Cluster 8	other,T3PKS,phenazine	5-acetyl-5,10-dihydrophenazine-1-carboxylic acid / endophenazine A	Other:Phenazine	100%
Cluster 9	NRPS,terpene	SCO-2138	RiPP	92%
Cluster 10	melanin	melanin	Other	100%
Cluster 11	siderophore	desferrioxamin B / desferrioxamine E	Other	66%
Cluster 12	T3PKS	germicidin	Other	100%
Cluster 13	PKS-like,butyrolactone	coelimycin P1	Polyketide:Modular type I	12%
Cluster 14	NRPS	diisonitrile antibiotic SF2768	NRP	66%
Cluster 15	lanthipeptide-Class I			
Cluster 16	linaridin	legonaridin	RiPP	66%
Cluster 17	NRPS	phosphonoglycans	Saccharide	3%
Cluster 18	amglyccycl	acarbose	Saccharide	7%
Contig 2 (GenBank accesion numbre JAGTPG010000001)				
Cluster 1	NRPS,other	himastatin	NRP	36%
Cluster 2	terpene	hopene	Terpene	53%
Cluster 3	hglE-KS	kanamycin	Saccharide	2%
Cluster 4	siderophore			
Cluster 5	terpene	geosmin	Terpene	100%
Cluster 6	RiPP-like			
Cluster 7	ectoine	ectoine	Other	75%
Cluster 8	siderophore			
Cluster 9	T2PKS	spore pigment	Polyketide	58%

Table S2

Table S2. Genes predicted in PM130391, 130392, and 140293 biosynthet gene cluster and predicted function of the encoded enzymes.

Gene code	Predicted enzime activity
1- KEF29_00490	VOC family protein
2- KEF29_00495	TetR
3- KEF29_00500	MMPL family transporter
4- KEF29_00505	ABC transporter permease
5- KEF29_00510	ATP-binding cassette domain-containing protein
6- KEF29_00515	EXLDI protein
7- KEF29_00520	Amidinotransferase
8- KEF29_00525	Cytochrome P450
9- KEF29_00530	Cytochrome P450
10- KEF29_00535	Cytochrome P450
11- KEF29_00540	NRPS: A
12- KEF29_00545	Hypothetical protein
13- KEF29_00550	Mycofactocin-coupled SDR family oxidoreductase
14- KEF29_00555	NRPS: TE
15- KEF29_00560	MbtH family protein
16- KEF29_00565	KtzI L-ornithine N(5)-oxygenase
17- KEF29_00570	NRPS: C-A-T-TE
18- KEF29_00575	NRPS: A-NMT-T
19- KEF29_00580	E-TIGR01720-C
20- KEF29_00585	NRPS: A
21- KEF29_00590	NRPS: C
22- KEF29_00600	NRPS: A
23- KEF29_00605	NRPS: C
24- KEF29_00610	NRPS: C
25- KEF29_00615	NRPS: C-A-T-E-E
26- KEF29_00620	PKS: AT
27- KEF29_00625	PKS: TE
28- KEF29_00630	PKS: KS (Iterative)
29- KEF29_00635	LmbU Transcriptional regulator
30- KEF29_00640	KtzT Piperazic acid synthase/FMN-binding negative transcriptional regulator
31- KEF29_00645	NRPS: C*
32- KEF29_00650	Hypothetical protein
33- KEF29_00655	NRPS: A
34- KEF29_00660	NRPS: T-E-E
35- KEF29_00665	NRPS: C
36- KEF29_00670	NRPS: T
37- KEF29_00675	Halogenase
38-KEF29_00680	DUF1707 domain-containing protein
39- KEF29_00685	Transporter
40- KEF29_00690	Hopanoid C-3 methylase
41- KEF29_00695	Hypothetical protein

42- KEF29_00700	Aquaporin family protein
43- KEF29_00705	Hypothetical protein
44- KEF29_00710	Methyltransferase domain-containing protein

References

55. Blin, K.; Shaw, S.; Steinke, K.; Villebro, R.; Ziemert, N.; Lee, S.Y.; Medema, M.H.; Weber, T. antiSMASH 5.0: updates to the secondary metabolite genome mining pipeline. *Nucleic Acids Res* **2019**, *47*, W81-W87.

56. Kautsar, S.A.; Blin, K.; Shaw, S.; Navarro-Munoz, J.C.; Terlouw, B.R.; van der Hooft, J.J.J.; van Santen, J.A.; Tracanna, V.; Suarez Duran, H.G.; Pascal Andreu, V.; Selem-Mojica, N.; Alanjary, M.; Robinson, S.L.; Lund, G.; Epstein, S.C.; Sisto, A.C.; Charkoudian, L.K.; Collemare, J.; Linington, R.G.; Weber, T.; Medema, M.H. MIBiG 2.0: a repository for biosynthetic gene clusters of known function. *Nucleic Acids Res* **2020**, *48*, D454-D458.
